# Perinatal exposure to pesticides alters synaptic plasticity signaling and induces behavioral deficits associated with neurodevelopmental disorders

**DOI:** 10.1007/s10565-022-09697-2

**Published:** 2022-02-08

**Authors:** Esperanza López-Merino, María I. Cuartero, José A. Esteban, Víctor Briz

**Affiliations:** 1 Centro de Biología Molecular Severo Ochoa (CSIC-UAM), Madrid, Spain; 2 Neurovascular Pathophysiology Group, Centro Nacional de Investigaciones Cardiovasculares Carlos III (CNIC), Madrid, Spain

**Keywords:** Contaminants, Kinase signaling, Psychiatric disorders, Neurodevelopment, MAPK, mGluR LTD

## Abstract

Increasing evidence from animal and epidemiological studies indicates that perinatal exposure to pesticides cause developmental neurotoxicity and may increase the risk for psychiatric disorders such as autism and intellectual disability. However, the underlying pathogenic mechanisms remain largely elusive. This work was aimed at testing the hypothesis that developmental exposure to different classes of pesticides hijacks intracellular neuronal signaling contributing to synaptic and behavioral alterations associated with neurodevelopmental disorders (NDD). Low concentrations of organochlorine (dieldrin, endosulfan, and chlordane) and organophosphate (chlorpyrifos and its oxon metabolite) pesticides were chronically dosed ex vivo (organotypic rat hippocampal slices) or in vivo (perinatal exposure in rats), and then biochemical, electrophysiological, behavioral, and proteomic studies were performed. All the pesticides tested caused prolonged activation of MAPK/ERK pathway in a concentration-dependent manner. Additionally, some of them impaired metabotropic glutamate receptor-dependent long-term depression (mGluR-LTD). In the case of the pesticide chlordane, the effect was attributed to chronic modulation of MAPK/ERK signaling. These synaptic alterations were reproduced following developmental in vivo exposure to chlordane and chlorpyrifos-oxon, and were also associated with prototypical behavioral phenotypes of NDD, including impaired motor development, increased anxiety, and social and memory deficits. Lastly, proteomic analysis revealed that these pesticides differentially regulate the expression of proteins in the hippocampus with pivotal roles in brain development and synaptic signaling, some of which are associated with NDD. Based on these results, we propose a novel mechanism of synaptic dysfunction, involving chronic overactivation of MAPK and impaired mGluR-LTD, shared by different pesticides which may have important implications for NDD.

**Graphical abstract:**

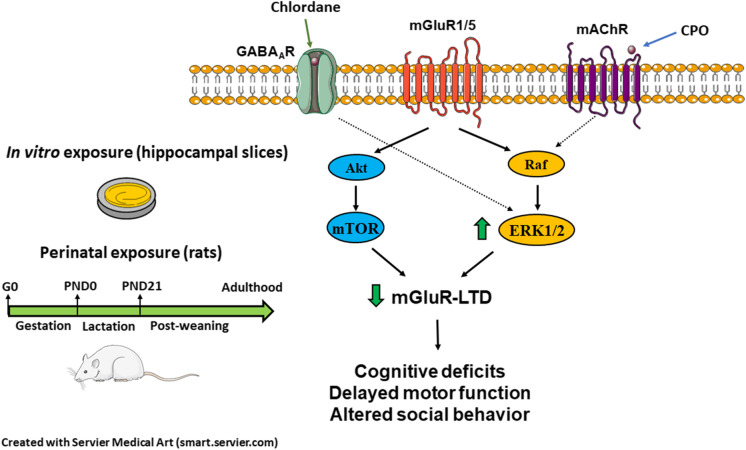

**Supplementary Information:**

The online version contains supplementary material available at 10.1007/s10565-022-09697-2.

## Introduction

Neurodevelopmental disorders include a wide range of genetic conditions that express characteristic converging phenotypes, including developmental motor delay, alterations in social and emotional behaviors, and cognitive deficits. Whilst the genetic contribution to neurodevelopmental disorders such as autism spectrum disorders (ASD) and intellectual disabilities has been extensively studied, genetic factors account only for a small fraction of cases and cannot explain the wide variation in the observed clinical features (Landrigan 2010). Inadvertent exposure to low concentrations of environmental contaminants such as pesticides has been associated with neurodevelopmental disorders affecting millions of children worldwide, in what has been called a silent pandemic (Grandjean et al. 2014; Grandjean and Landrigan 2006). In this line of evidence, an increasing number of animal and epidemiological studies indicate that prenatal and early postnatal exposure to organochlorine and organophosphate pesticides produce developmental neurotoxicity (Burke et al. 2017; Grandjean and Landrigan 2006) and may also increase the risk for ASD (Kalkbrenner et al. 2014; Rossignol et al. 2014; Shelton et al. 2012) and attention deficit hyperactivity disorder (ADHD) (Polańska et al. 2013; Roberts et al. 2019). However, the molecular mechanisms linking exposure to pesticides and neurodevelopmental disorders remain largely unexplored. Most of these chemicals are included in the Agency for Toxic Substances and Disease Registry 2019 Substance Priority List (https://www.atsdr.cdc.gov/spl/index.html#2019spl), including the organochlorines dieldrin, endosulfan and chlordane, as well as chlorpyrifos (CPF), an organophosphate. Furthermore, some of them have been listed as persistent organic pollutants under the Stockholm Convention (http://www.pops.int), and hence their use and production are banned or restricted globally. Yet, human populations are still exposed to many of these contaminants, as they bioaccumulate in fatty tissues leading to escalating dietary exposure along the trophic chain (González-Alzaga et al. 2018; Hertz-Picciotto et al. 2018; Junqué et al. 2017).

Alterations in synaptic structure and function are believed to play a major role in the pathophysiology of neurodevelopmental disorders (Bagni and Zukin 2019; Forrest et al. 2018). Particularly, synaptic plasticity events such as long-term potentiation (LTP) and long-term depression (LTD) are typically affected in genetic animal models of ASD and intellectual disability (D’Antoni et al. 2014; Knafo and Esteban 2017). Two major signaling pathways, namely protein kinase B (Akt)/mammalian target of rapamycin (mTOR) and mitogen-activated protein kinase (MAPK)/extracellular regulated kinase (ERK), are known to regulate synaptic plasticity, including LTP and metabotropic glutamate receptor-dependent long-term depression (mGluR-LTD) in the CA1 area of the hippocampus (Hoeffer and Klann 2010; Sanderson et al. 2016). These signaling cascades are key upstream regulators of dendritic protein synthesis, an event required for the maintenance of both LTP and mGluR-LTD that is dysregulated in several animal models of ASD (Borrie et al. 2017; Huber et al. 2015). However, mechanisms linking alterations in kinase signaling, local mRNA translation, and synaptic physiology with behaviors relevant to neurodevelopmental disorders still remain to be elucidated. Also, although studies reported synaptic and behavioral deficits associated with exposure to organochlorine and organophosphate pesticides both in animal models and humans (Albertson et al. 1997; Grandjean et al. 2014; Olmos et al. 2009; Schantz and Widholm 2001), it is not clear how they could contribute to the molecular, cellular, and behavioral abnormalities of neurodevelopmental disorders. Here, we aimed to shed light into these issues by studying the developmental effects of exposure to some of these pesticides on synaptic function and protein signaling using the hippocampus as a model system for synaptic physiology, and the rat as animal model for the study of behaviors relevant to neurodevelopmental disorders.

## Methods

### Animals and ethics statement

All biosafety procedures and animal care protocols used were approved by the bioethics committee of UAM and CSIC and the local regulatory authorities, and performed according to Spanish (RD 53/2013, 32/2007) and EU guidelines set out in the European Community Council Directives (86/609/EEC). All personnel involved in the animal care and experimentation was appropriately trained according to FELASA standards. Animal’s health and welfare were monitored by a designated veterinarian. Animals were housed in cages that meet all regulatory requirements and the animal rooms have a management system that controls temperature, light, and humidity. Food and water were provided ad libitum and shredded tissue paper was used as basic enrichment for nest building. Wistar rats of both genders were used for all experiments.

### Preparation of hippocampal slices

Organotypic hippocampal slice cultures were prepared from postnatal day (PND) 5–7 rats as previously described (Brachet et al. 2015). Acute hippocampal slices were prepared from adolescent (PND14-30) and adult (8–11 weeks old) female and male rats as described previously (Draffin et al. 2021), and kept at 32 °C for 1 h in artificial cerebrospinal fluid (aCSF, 119 mM NaCl, 2.5 mM KCl, 2.5 mM CaCl_2_, 1.25 mM MgCl_2_, 26 mM NaHCO_3_, 1 mM NaH_2_PO_4_, 11 mM glucose, pH 7.4, and osmolarity adjusted to 290 ± 5 mOsm) before use.

### Electrophysiology

Whole-cell voltage-clamp and current-clamp recordings were obtained from CA1 pyramidal neurons in organotypic rat hippocampal slice cultures, as previously described (Draffin et al. 2021). For details, see Supplementary Information.

Field excitatory postsynaptic potentials (fEPSPs) were recorded at 30 °C from acute slices with glass electrodes (0.2–0.8 MΩ) filled with aCSF, and placed in CA1 *stratum radiatum* using different stimulation intensities to generate an input–output curve of fEPSP initial slope, a more reliable measure of fEPSP than its peak amplitude (Hawkins et al. 2017). This curve was also used to set the baseline of fEPSP slope value at ≈70% of the maximum response. mGluR-LTD was induced by paired pulse low frequency stimulation (PP-LFS, 900 paired pulses, separated by 50 ms at 1 Hz) in the presence of 100 µM (2R)-amino-5-phosphonovaleric acid (AP5) and 100 μM picrotoxin (PTX).

### Chemical LTP/LTD induction and western blotting

Cultured hippocampal slices were transferred to a submersion-type holding chamber containing aCSF gassed with 5% CO_2_/95% O_2_ at 29 °C for 30 min before the induction of plasticity protocols. To induce LTP chemically, a cocktail containing 50 µM forskolin, 0.1 µM rolipram, and 100 µM PTX was prepared in a Mg-free aCSF and incubated for 15 min (cLTP); or a tetraethylammonium (TEA, 25 mM) solution was incubated for 10 min. For induction of LTD, the mGluR1/5 agonist DHPG (100 µM, 10 min) was used. After washing out the drugs, slices were homogenized in lysis buffer (150 mM NaCl, 50 mM Tris–HCl pH 7.4, 1% Triton X-100) containing protease and phosphatase inhibitor cocktails (complete mini EDTA-free and phosSTOP, Roche), and processed for western blot, as previously described (Briz and Baudry 2014). Hippocampal samples from adult rats following perinatal exposure to pesticides were similarly processed for western blotting. See Supplementary Information for details on procedures and antibodies used.

### Puromycin labelling

Protein synthesis was measured by using puromycin labelling as previously described (Briz et al. 2017), with minor modifications. For details, see Supplementary Information.

### Behavior

Animals were housed in groups of 2–4 per cage in a flow cabinet with 12-/12-h dark–light cycle. Each animal was handled for 2 min in the experimental room on each of the 3 days before starting the experiments. In all tests, female and male rats were analyzed separately and their data generally pooled together, unless sex-differences were observed. For a detailed description of the procedures and tests, see Supplementary Information. Table S3 lists number of animals and litters used in behavior.

### Proteomics

Proteomics was performed by liquid chromatography electrospray ionization and tandem mass spectrometric (LC–ESI–MS/MS) analysis. For a detailed methodological description on sample processing and data analysis, see Supplementary Information.

### Statistical analyses

Unless otherwise indicated, results were represented as means ± standard error of the mean (SEM). The number of independent experiments (*n*) usually refers to the number of animals used, except for electrophysiology experiments, where it refers to the number of neurons (for patch-clamp recordings) or slices (for field recordings), obtained from at least 3 animals. For biochemical experiments, *n* refers to the number of independent cultured batches (each batch was a combination of slices from 2 rat pups). The cut-off value for statistical significance was set at *P* < 0.05. For experiments where only two groups were compared (some in vivo experiments), we used the two-tail *t*-test or Mann–Whitney test to determine statistical significance. When more than 2 groups were compared, we used one-way or two-way analyses of variance (ANOVA) followed by Dunnett’s or Bonferroni’s post-test analysis, respectively. Graphpad Prism 5.01 was used to run the statistics. A complete report of the statistical values and tests used for this work can be found in Table S4.

## Results

### Pesticides chronically activate MAPK/ERK signaling in hippocampal slice cultures

**Fig. 1:**
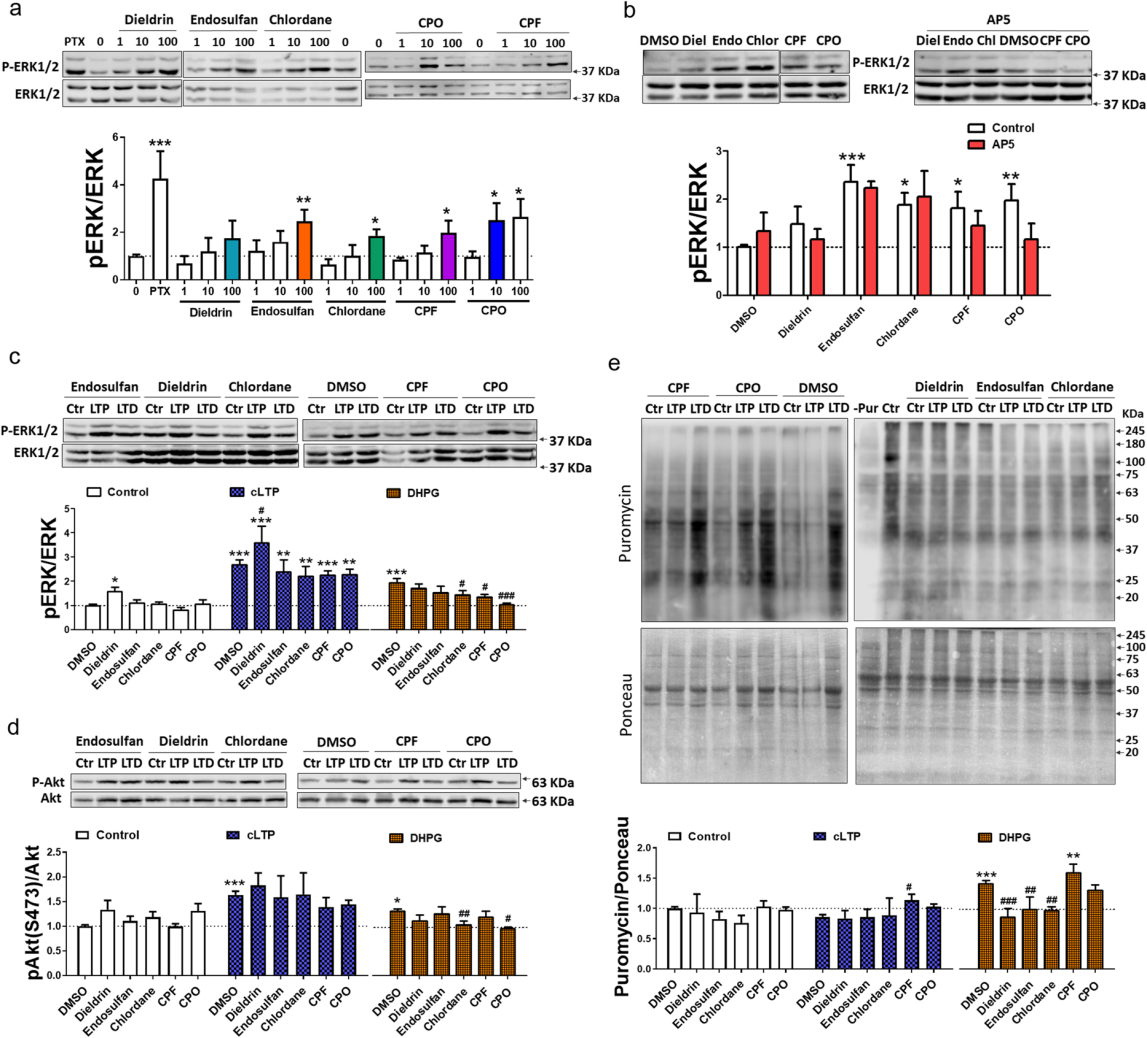
Chronic effects of pesticides on plasticity-regulated signaling. **a** Western blots of P-ERK1/2 vs. total ERK1/2 in organotypic hippocampal slices exposed chronically to DMSO or to 1–100 nM of pesticides. Mean ± SEM, *n* = 3–6, *P < 0.05, **P < 0.01 vs. DMSO, one-way ANOVA + Dunnett’s post-test. **b** Western blots of P-ERK1/2 vs. total ERK1/2 in organotypic hippocampal slices exposed chronically to contaminants in the absence or presence of 100 µM AP-5. Mean ± SEM, *n* = 3–9, *P < 0.05, **P < 0.01, ***P < 0.001 vs. DMSO, two-way ANOVA + Bonferroni’s post-test. **c**, **d** Western blots of P-ERK1/2 vs. total ERK1/2 and P-Akt vs. total Akt 15 min after chemical LTP (LTP) and DHPG (LTD) treatments in slices exposed chronically to pesticides. Mean ± SEM, *n* = 4–12, *P < 0.05, **P < 0.01, ***P < 0.001 vs. control, ^**#**^*P* < 0.05, ^**###**^*P* < 0.001 vs. LTP/DHPG alone, two-way ANOVA + Bonferroni’s post-test. **e** Puromycin labelling after chemical LTP (LTP) and DHPG (LTD) treatments in slices exposed chronically to pesticides. Slices non-treated with puromycin (-Pur) were used as negative control. Mean ± SEM, *n* = 3–8, **P < 0.01, ***P < 0.001 vs. control, ^**#**^*P* < 0.05, ^**##**^*P* < 0.01, ^**###**^*P* < 0.001 vs. DHPG alone, two-way ANOVA + Bonferroni’s post-test

Alterations in Akt/mTOR and MAPK/ERK signaling pathways are common pathological features of neurodevelopmental disorders, including non-syndromic, idiopathic forms of autism (Borrie et al. 2017; Huber et al. 2015). We then assessed the effects of chronic (from day in vitro- DIV-0 to DIV 7–10) exposure to several pesticides in organotypic hippocampal cultures, monitoring the phosphorylation state of different proteins of these signaling cascades by western blot. The chemicals selected were three organochlorine pesticides (dieldrin, endosulfan, and chlordane) and two organophosphate pesticides (CPF and its metabolite chlorpyrifos-oxon [CPO]), based on their developmental neurotoxicity (Grandjean et al. 2014; Grandjean and Landrigan 2006; Shelton et al. 2012) and their proposed association with neurodevelopmental disorders (Kalkbrenner et al. 2014; Polańska et al. 2013; Roberts et al. 2019; Rossignol et al. 2014; Shelton et al. 2012). We used a range of concentrations (1–100 nM) similar to those typically used in long-term, in vitro toxicological studies (Briz et al. 2012; Prendergast et al. 2007), which are relevant and translatable to human exposure levels (González-Alzaga et al. 2018; Hertz-Picciotto et al. 2018; Junqué et al. 2017). None of the chemicals significantly alter Akt (neither at T308 nor S473), mTOR, or S6 phosphorylation at any of the concentrations tested (Fig. S1). In marked contrast, all the pollutants produced a concentration-dependent increase in ERK phosphorylation (Fig. 1a). Of note, these effects were not reproduced following acute (2 h) exposure to 100 nM of any chemical (Fig S2), indicating that the effects on MAPK signaling are due to chronic modulation of the pathway. As a reference, both acute and chronic enhancements of neuronal activity by exposure to the γ-aminobutyric acid A receptor (GABA_A_R) antagonist picrotoxin (PTX, 100 µM), a drug with a similar pharmacological profile as organochlorines (Vale et al. 2003), produced a dramatic activation of MAPK/ERK but not Akt/mTOR/S6 signaling (Fig. 1a and S2).

Based on these results, we selected the lowest observed effect concentration (or 100 nM if no significant effect was observed) for subsequent experiments (Fig. 1a, colored bars). In order to address whether these effects involved synaptic mechanisms, we chronically exposed slice cultures to the contaminants in the presence of glutamate receptor antagonists. Indeed, co-exposure with the N-methyl-D-aspartate receptor (NMDAR) antagonist AP5 partially restored the levels of phospho-ERK (p-ERK) to control conditions in slices treated with CPO (significant interaction, *P* < 0.05, two-way ANOVA), while it did not modify the effects of organochlorines or CPF (Fig. 1b). The increase in ERK phosphorylation induced by organochlorines was also insensitive to the mGluR1/5 antagonist MCPG (Fig. S3). In addition, chronic exposure to different classes of pesticides significantly increased ERK activity in membrane fractions (*P* = 0.008, one-way ANOVA), and a similar trend was observed in the cytosol (Fig. S4a). In contrast, no effects were observed in the phosphorylation state of p38, another MAPK. In summary, the pesticides tested chronically modulate MAPK/ERK but not Akt/mTOR/S6 or MAPK/p38 signaling in the hippocampus, although they seem to exert these effects via distinct mechanisms.

### Chronic exposure to pesticides suppresses mGluR-LTD signaling

Akt/mTOR and MAPK/ERK pathways have been shown to be required for different forms of synaptic plasticity, including LTP and mGluR-LTD (Hoeffer and Klann 2010; Sanderson et al. 2016). To address whether exposure to pesticides may affect plasticity-induced kinase signaling, we determined changes on these pathways following synaptic stimulation with different chemical protocols of LTP and LTD in slices chronically treated with pesticides. For these experiments, slices were first washed-out in aCSF for 40–45 min to remove the contaminants before plasticity induction. Under these conditions, with the exception of dieldrin, the enhanced p-ERK signal was no longer observed (Fig. 1c; white columns), which indicates that there is a rapid recovery of basal MAPK activity during the washing out period before stimulation. In this manner, we were able to evaluate persistent effects of the contaminants on the signaling cascades triggered at the moment of plasticity induction. A cocktail of forskolin, rolipram, and PTX, widely used to induce a chemical form of LTP (cLTP) (Otmakhov 2004), stimulated both Akt and ERK phosphorylation in slices treated with DMSO (Fig. 1c,d; blue columns), a result consistent with previous findings (Gobert et al. 2008). Similar effects were generally observed in slices treated with the different pesticides. We also tested the effects of the potassium channel blocker TEA, which induces an NMDAR-independent, but MAPK-dependent form of LTP (TEA-LTP) (Kanterewicz et al. 2000). This protocol induced a robust activation of Akt and ERK in control slices and also in those treated with pollutants (Fig. S5). We next tested the effects of the mGluR1/5 agonist DHPG, which induced an increase in both Akt and ERK phosphorylation in DMSO-treated slices. Notably, the effects on Akt and ERK were significantly blocked in slices treated with chlordane and CPO (Fig. 1c, d; orange columns).

Both LTP and mGluR-LTD are known to engage mRNA translation mechanisms, also when induced chemically (Gobert et al. 2008; Waung and Huber 2009). To further study the chronic effects of pesticides on plasticity-related signaling, we used puromycin labeling, a non-radioactive method to monitor de novo protein synthesis (Schmidt et al. 2009). Under control conditions, no significant difference in basal translation was found among the pollutants as compared to DMSO-treated slices (Fig. 1e; white columns). Surprisingly, no changes were observed on puromycin labeling 15 min following induction of cLTP (Fig. 1e; blue columns). However, while DHPG caused a significant increase in translation in slices treated with DMSO or CPF, this effect was completely abolished in those treated with organochlorines and partially blocked in CPO-treated slices (Fig. 1e; orange columns). These results are consistent with our previous findings on kinase activation, and indicate that pesticides selectively affect synaptic signaling related to mGluR-LTD.

### Pesticides impair mGluR-LTD but not LTP or NMDAR-LTD

**Fig. 2:**
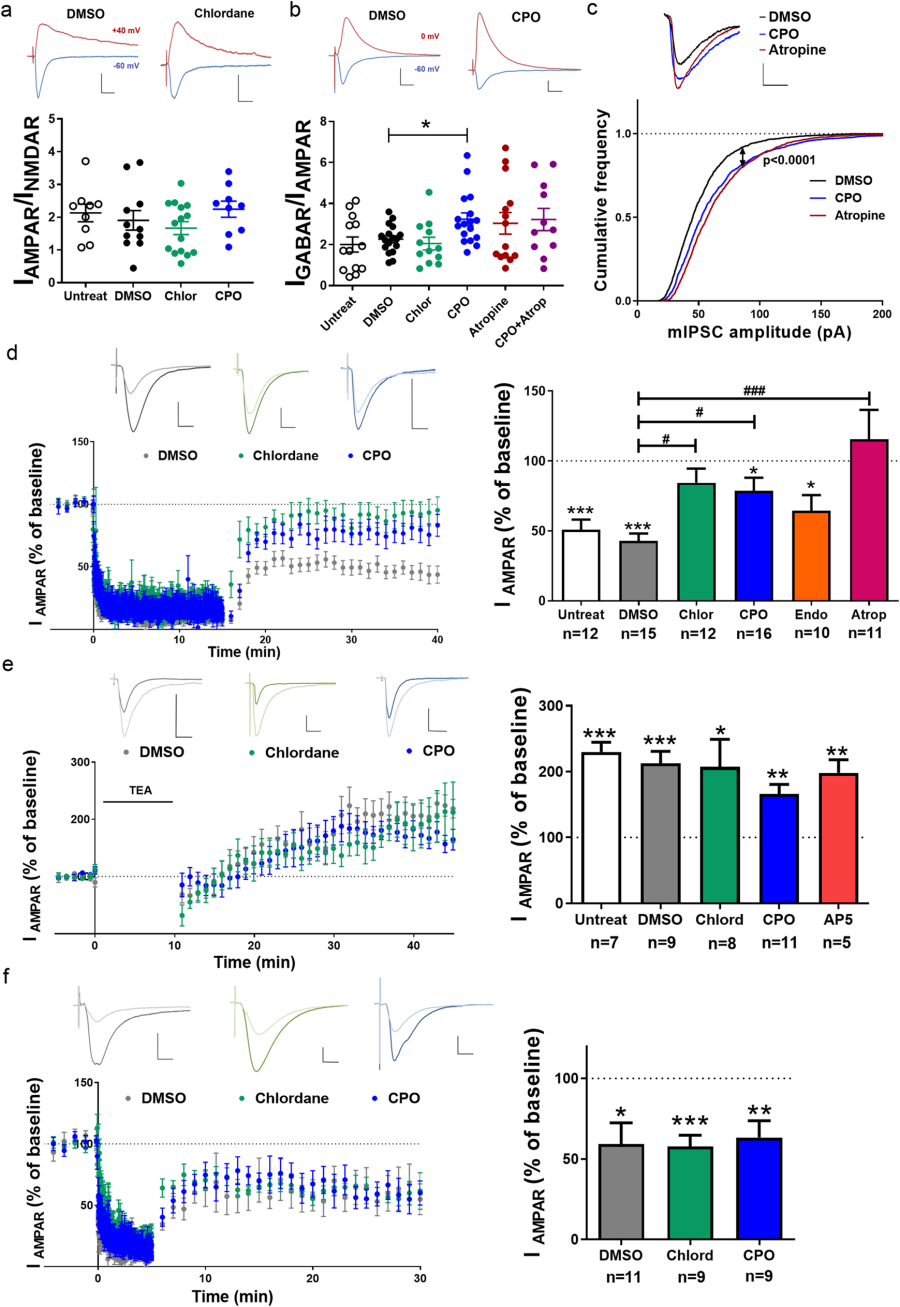
Chronic effects of pesticides on basal transmission and synaptic plasticity. Patch**-**clamp recordings of CA1 pyramidal neurons from slices exposed chronically to DMSO, pesticides, and/or 100 nM atropine. Non-treated slices (untreat) were used as control. **a** Representative traces (above) and quantification (below) of AMPAR vs. NMDAR currents (I). Mean ± SEM, *n* = 9–15. Scale bars = 40 pA/20 ms. **b** Representative traces (above) and quantification (below) of GABA_A_R vs. AMPAR currents (I). Mean ± SEM, *n* = 9–17, *P < 0.05, one-way ANOVA + Dunnett’s post-test. Scale bars = 100 pA/20 ms. **c** Representative traces (above) and cumulative frequency distribution (below) of miniature inhibitory postsynaptic currents (mIPSC) amplitude. *n* = 13–20 neurons and 1270–1599 events; ***P < 0.001 for CPO and atropine as compared to DMSO, Kolmogorov–Smirnov test. Scale bars = 20 pA/10 ms. **d** Representative traces (above) and quantification (right) of AMPAR currents before (baseline, dark line) and 35–40 min after (light line) induction of mGluR-LTD. Results are expressed as % compared to baseline responses. *P < 0.05, ***P < 0.001 vs. baseline (one-sample *t*-test); ^**#**^*P* < 0.05, ^**##**^*P* < 0.01 vs. DMSO (one-way ANOVA + Dunnett’s post-test). Scale bars = 40 pA/20 ms. **e** Representative traces (above) and quantification (right) of AMPAR currents before (baseline, dark line) and 40–45 min after (light line) induction of TEA-LTP. Slices were treated with 100 µM AP-5 only during the induction. Results are expressed as % compared to baseline responses. *P < 0.05, **P < 0.01, ***P < 0.001 vs. baseline (one-sample *t*-test). Scale bars = 50 pA/20 ms. **f** Representative traces (above) and quantification (right) of AMPAR currents before (baseline) and after (25–30 min) induction of NMDA-LTD. Results are expressed as % compared to baseline responses. *P < 0.05, **P < 0.01, ***P < 0.001 vs. baseline (one-sample *t*-test). Scale bars = 40 pA/20 ms

Based on these results, we selected chlordane and CPO (those with the most notable phenotypes on activity-induced signaling) to test their effects on synaptic physiology. Endosulfan was included in some experiments as a negative control. First, we analyzed the effects of chronic exposure to these pollutants on basal synaptic transmission in CA1 pyramidal neurons by whole-cell voltage-clamp recordings. Again, contaminants were washed-out from the slices before starting the recordings, to assess their persistent effects. Ratio of α-amino-3-hydroxy-5-methyl-4-isoxazolepropionic acid receptor (AMPAR)/NMDAR synaptic currents in slices treated with contaminants was similar compared to DMSO-treated or non-treated slices (Fig. 2a). Consistent with this, the levels of the two main AMPAR subunits, GluA1 and GluA2, and the obligatory NMDAR subunit GluN1 in membranes from hippocampal homogenates were not statistically different across the different treatment groups (Fig. S4b-d). Interestingly, the ratio of GABA_A_R/AMPAR currents was significantly increased in CPO-treated slices as compared to DMSO (Fig. 2b; *P* = 0.0087, one-way ANOVA). This result suggests that CPO may produce an imbalance between excitatory and inhibitory transmission.

To explore the molecular mechanisms underlying the effects of CPO on basal transmission, we chronically treated slices with the muscarinic receptor antagonist atropine (100 nM) alone and together with CPO, as this pesticide is known to modulate cholinergic transmission (Huff et al. 1994; Liu et al. 2002). Surprisingly, we found that atropine increased GABA_A_R/AMPAR currents to a similar extent as CPO, and this effect was not further enhanced when the two chemicals were present (Fig. 2b), possibly indicating that they share a common mechanism to increase GABA transmission. In order to directly assess inhibitory synaptic transmission, we analyzed miniature inhibitory postsynaptic currents (mIPSC) in hippocampal slices treated with either CPO or atropine. Indeed, both drugs similarly enhanced mIPSC amplitude as compared to DMSO-treated neurons (Fig. 2c). We also found that holding currents, resting membrane potential, and input resistance were equivalent for all treatment groups (Fig. S6a-c), indicating that passive membrane properties were unaffected by the chemicals. In contrast, membrane capacitance was slightly, but significantly reduced in CPO-treated neurons as compared to DMSO- and chlordane-treated cells (Fig. S6d). We further explored whether neuronal excitability was affected by the pesticides. Action potential (AP) threshold, post-AP hyperpolarization, and firing frequency in CPO- or chlordane-treated slices were not significantly different from DMSO-treated slices (Fig. S6e-g), although there were statistically significant differences between the two pesticides on firing frequency. Additionally, no differences were found in spontaneous activity among the tested groups (Fig. S6h).

Next, we tested the effects of pesticides on different synaptic plasticity paradigms, including mGluR-LTD, LTP, and NMDAR-LTD. In untreated or DMSO-treated slices, PP-LFS in the presence of AP5 induced a robust mGluR-LTD, as expected (Kemp and Bashir 1999; Zhu et al. 2017). In contrast, this form of LTD was significantly impaired in slices exposed to chlordane and CPO, while it remained expressed in slices treated with endosulfan (Fig. 2d). In slices treated chronically with atropine, mGluR-LTD was completely suppressed, hence again mimicking the effects of CPO.

Previous work has shown that long-term exposure to organochlorines downregulates the expression of mGluR5 in cultured neurons (Briz et al. 2009). Therefore, the observed effects on mGluR-LTD could be due to alterations in mGluR expression and/or localization. However, neither the membrane nor the cytosolic levels of mGluR1/5 were affected by exposure to the different pollutants (Fig S4c), suggesting that their mechanism of mGluR-LTD disruption may lie downstream of mGluRs. Similarly, the levels of other synaptic proteins, including Homer-1, PSD-95, and SynGAP, remained unchanged by the treatments (Fig S4d).

We next tested the effects of these two pesticides on TEA-LTP, an NMDAR-independent form of LTP (Hanse and Gustafsson 1994; Kanterewicz et al. 2000). As expected, TEA-LTP was still expressed in the presence of the NMDAR antagonist AP5, and exposure to chlordane or CPO did not significantly affect it as compared to DMSO-treated slices (Fig. 2e). Likewise, none of the pesticides affected NMDAR-LTD as compared to DMSO (Fig. 2f).

### Restoring ERK activity rescues mGluR-LTD impairment by chlordane

**Fig. 3:**
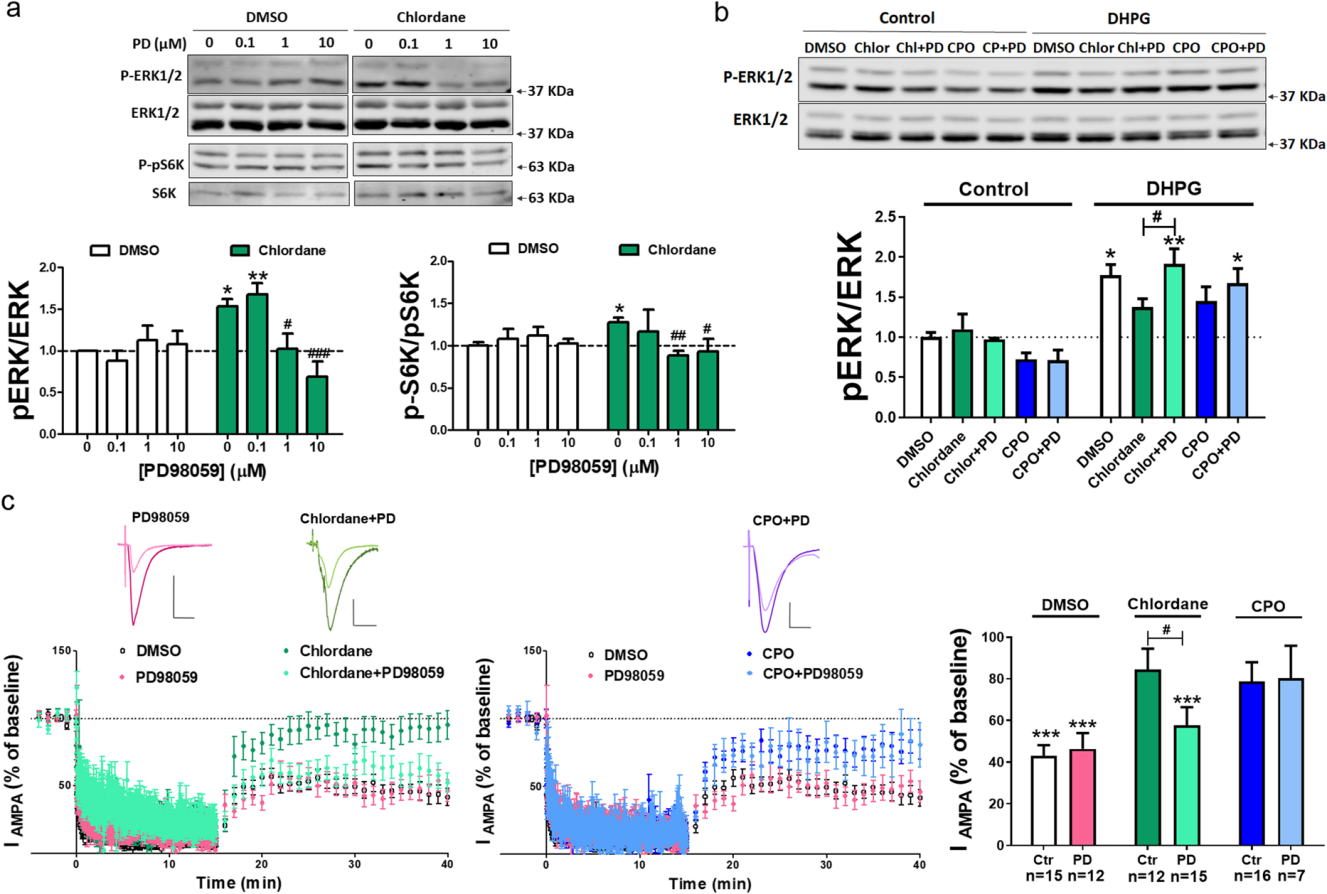
Chronic MAPK inhibition restores mGluR-LTD in chlordane-treated slices. **a** Western blots of P-ERK1/2 vs. total ERK1/2 and P-S6K vs. total S6K in organotypic hippocampal slices exposed chronically to 100 nM chlordane in the absence or presence of different concentrations of PD98059 (PD). Mean ± SEM, *n* = 4–8, *P < 0.05, **P < 0.01 vs. DMSO; ^**#**^*P* < 0.05, two-way ANOVA + Bonferroni’s post-test. **b** Western blots of P-ERK1/2 vs. total ERK1/2 in organotypic hippocampal slices exposed chronically to 100 nM chlordane or 10 nM CPO in the absence or presence of 1 µM PD 15 min after DHPG treatment. Mean ± SEM, *n* = 3–8, *P < 0.05, **P < 0.01 vs. control, ^**#**^*P* < 0.05, ^**##**^*P* < 0.01, ^**###**^*P* < 0.001 vs. chlordane alone, two-way ANOVA + Bonferroni’s post-test. **c** Representative traces (above) and quantification (right) of AMPA currents before (baseline) and after (35–40 min) induction of mGluR-LTD in slices treated chronically with 100 nM chlordane or 10 nM CPO in the presence of 1 µM PD. ***P < 0.001 vs. baseline, one-sample *t*-test; ^**#**^*P* < 0.05, two-way ANOVA + Bonferroni’s post-test. Scale bars = 40 pA/20 ms

Genetic overactivation of MAPK/ERK pathway has been shown to specifically impair mGluR-LTD but not other forms of plasticity in the hippocampus (Schreiber et al. 2017). We hypothesized that blockade of mGluR-LTD caused by pesticide exposure could result from chronic elevation of ERK activity. To address this question, we aimed at normalizing ERK signaling in treated slices by using sub-saturating concentrations of a MAPK inhibitor along with the pesticides. Chronic treatment with 1–10 µM PD98059 normalized basal ERK activity in chlordane-treated slices (Fig. 3a; green columns). Similar results were obtained for S6 Kinase (S6K, T421/S424), a substrate of ERK involved in translational regulation. Importantly, PD98059 did not affect basal ERK or S6K phosphorylation at the concentrations tested in DMSO-treated slices (Fig. 3a; white columns).

We next asked if restoring basal MAPK activity would render the slices again sensitive to stimulation by mGluR activation. Consistent with our previous results (Fig. 1c), chronic exposure to chlordane reduced DHPG-induced ERK activation. Importantly, concomitant treatment of chlordane with 1 µM PD98059 restored DHPG-induced stimulation of MAPK activity to control (DMSO) levels (Fig. 3b). A similar trend was found for CPO plus PD98059, but the results were not statistically different as compared to CPO alone. It is important to note that the basal levels of p-ERK (in control slices) were not statistically different across the treatment groups.

These results strongly suggest that chronic overactivation of the MAPK pathway makes it unresponsive to later stimulation, and importantly, normalizing this pathway pharmacologically with subsaturating concentrations of a MAPK inhibitor renders it again sensitive to mGluR stimulation. We then tested whether rescuing biochemical MAPK responsiveness may also restore mGluR-dependent plasticity. Indeed, chronic attenuation of MAPK activity rescued mGluR-LTD in chlordane-treated slices. However, PD98059 treatment failed to rescue mGluR-LTD in slices treated with CPO. To note, PD98059 treatment on its own had no effect on mGluR-LTD as compared to DMSO (Fig. 3c). Therefore, taken all together, our findings suggest that chlordane and CPO modulate mGluR-LTD via MAPK-dependent and MAPK-independent mechanisms, respectively.

### Perinatal exposure to pesticides impairs hippocampal mGluR-LTD

**Fig. 4:**
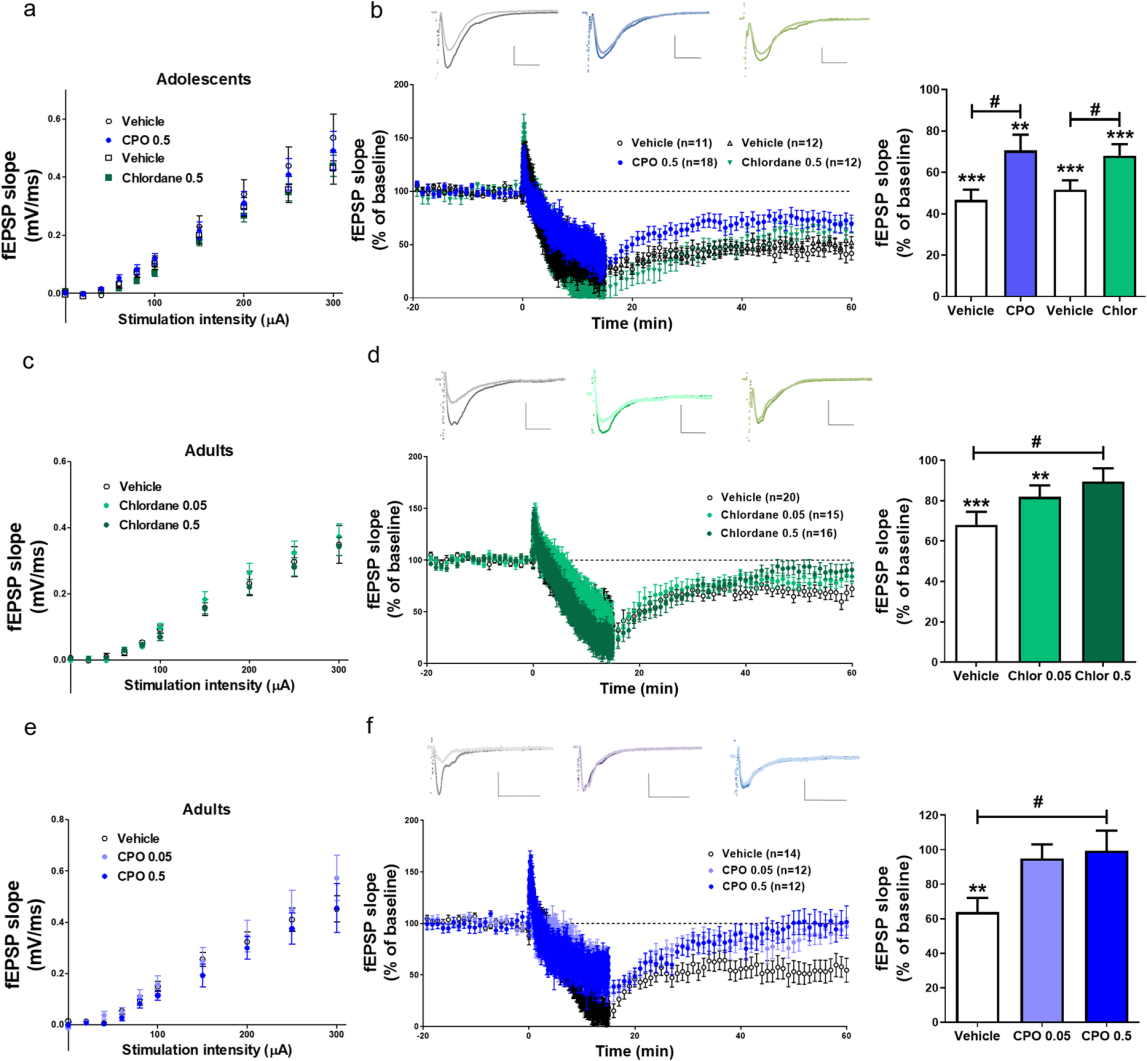
Perinatal exposure to chlordane and CPO impairs mGluR-LTD. Field recordings of CA1 area of hippocampus in acute slices from 0.05-0.5 mg/Kg/day CPO-, chlordane-, and DMSO-treated (vehicle) rats. **a**, **c**, **e** Input/output curves of field excitatory postsynaptic potentials (fEPSP) slope. Mean ± SEM, *n* = 14–17. **b**, **d**, **f** Representative traces and quantification of fEPSP slope before (baseline) and after (55–60 min) induction of mGluR-LTD in PND14-30 juvenile (**b**) and PND55-77 adult (**d**, **f**) rats. Results are expressed as % of fEPSP slope compared to baseline responses. Scale bars = 0.2 mV/ms /20 ms. **P < 0.01, ***P < 0.001 vs. baseline (one-sample *t*-test); **b** Mean ± SEM, *n* = 10–18, ^**#**^*P* < 0.05, two-tailed *t*-test. **d**, **f** Mean ± SEM, *n* = 12–18, ^**#**^*P* < 0.05, one-way ANOVA + Dunnett’s post-test

After establishing the signaling and synaptic alterations induced by chlordane and CPO on hippocampal slice cultures, we aimed to assess the relevance of these observations ex vivo. To this end, we first evaluated the effects of perinatal exposure to low doses of these pesticides on synaptic function at different developmental stages. Rats were exposed to chlordane or CPO (at 0.05–0.5 mg/kg/day) from gestational day 0, and at PND14–30 acute hippocampal slices were prepared from offspring and field recordings obtained from CA1 area. Basal synaptic transmission as measured by input/output curves of field potentials was similar across the different treatment groups (Fig. 4a). We then tested mGluR-LTD using the same induction protocol as described before. As shown in Fig. 4b, robust mGluR-LTD was observed in slices from vehicle-treated animals. In contrast, mGluR-LTD was significantly attenuated in slices from both CPO- and chlordane-treated rat pups (Fig. 4b). Similar to our previous results with younger rats, both pesticides suppressed mGluR-LTD in 8-–11-week-old adult rats in a dose-dependent manner (Fig. 4d, f) without affecting basal transmission (Fig. 4c, e). It is worth noting that rats exposed to pesticides (0.5 mg/kg/day) displayed overall normal gross brain morphology and similar hippocampal and cortical size as compared to vehicle-treated rats (Fig S7), albeit a trend towards increased hippocampal area was observed in rats exposed to CPO (Fig S7g, h). We also found that perinatal exposure to chlordane and CPO increased ERK phosphorylation in hippocampal homogenates from adult rats as compared to vehicle-treated rats, although the effect was observed in male but not in female rats (Fig. S8a).

In order to assess whether the effects of CPO and chlordane had a neuroinflammatory component, including astrogliosis and microgliosis, we determined the levels of GFAP and Iba1, respectively, in hippocampal lysates by immunoblot. Perinatal exposure to chlordane decreased GFAP levels in males but not in females, whereas it did not significantly affect the expression of the microglial marker Iba1 (Fig. S8b). In contrast, CPO exposure significantly increased Iba1 levels without affecting those of the astrocytic marker GFAP (Fig. S8b). These results suggest that the pesticides studied may have different effects on neuroinflammation in the hippocampus.

### Perinatal pesticide exposure causes motor and cognitive deficits

**Fig. 5:**
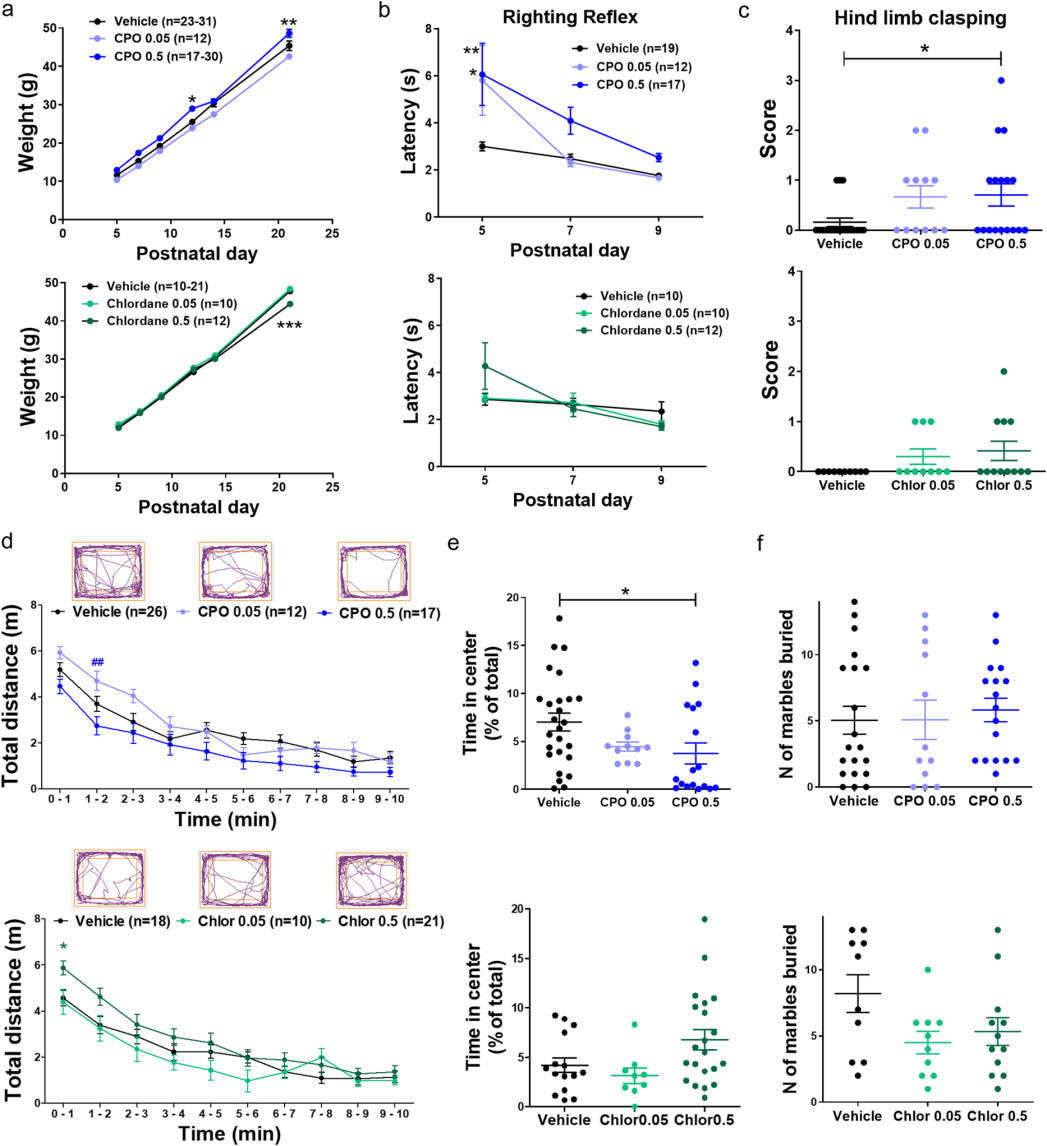
Perinatal exposure to pesticides alters motor development and locomotor activity. **a** Somatic growth of rat pups as measured by weight gain during the lactation period. *P < 0.05, **P < 0.01, ***P < 0.001 vs. vehicle, two-way ANOVA + Bonferroni’s post-test. **b** Latency to righting all four limbs during the first days of postnatal period. Mean ± SEM, values from 3 consecutive trials were averaged for each rat pup. *P < 0.05, **P < 0.01 vs vehicle, two-way ANOVA + Bonferroni’s post-test. **c** Hind limb clasping score at weaning (PND21), measured as previously described (Dodge et al. 2020). *P < 0.05, one-way ANOVA + Dunnett’s post-test. **d** Total distance travelled in the open field test divided by 1-min intervals. *P < 0.05 vs. vehicle, **P < 0.01 vs. CPO 0.5, two-way ANOVA + Bonferroni’s post-test. **e** Time in the center of the arena in the open field test. *P < 0.05, one-way ANOVA + Dunnett’s post-test. **f** Number of marbles buried during a 30-min period

We next wanted to explore whether these alterations in synaptic function were accompanied by behavioral phenotypes related to neurodevelopmental disorders. We found that perinatal exposure to CPO slightly increased pup weight at the highest dose (0.5 mg/kg/day; Fig. 5a) and significantly enhanced righting reflex latency in a dose-dependent manner (Fig. 5b). Of note, the deleterious effects on righting reflex were more evident in females compared to male rats (Fig S9a). In addition, we observed hind limb clasping in circa 50% of CPO-treated pups when subjected to tail suspension at weaning, while only 15% of the vehicle-treated animals exhibited it (Fig. 5c). Noteworthy, all the animals that scored 2 or higher in this test were CPO-treated males (Fig S9b). In contrast, perinatal exposure to chlordane did not affect righting reflex or hind limb clasping, but significantly decreased pup weight at weaning (PND21) at the highest dose tested (Fig. 5a–c, lower plots).

CPO-treated offspring had overall normal locomotor activity in the open field test as compared to vehicle-treated rats, albeit statistical differences were found among the two CPO groups at short time points (Fig. 5d). On the other hand, high-dose chlordane-treated rats showed hyperactivity at the beginning of the test but then habituated normally to the novel environment, a result consistent with previous work (Al-Hachim and Al-Baker 1973). This test also revealed that CPO-treated rats spent significantly less time in the center of the arena compared to vehicle-treated rats, suggesting increased anxiety in these animals. In contrast, chlordane-treated rats did not show thigmotaxis in the open field test (Fig. 5e). Compulsivity and repetitive behaviors were assessed with the marble burying test. However, neither CPO- nor chlordane-treated rats show any phenotype in this test (Fig. 5f, Fig. S10).

We used the maternal homing test, which has been previously utilized to study ASD-related behaviors, to evaluate social and olfactory discrimination in neonatal rats (Batista et al. 2019). In this test, animals were isolated from their mother at PND15 and placed on a separate cage containing bedding from their home cage in one corner and fresh bedding on the opposite corner, and allowed to freely explore the arena. The majority of vehicle and CPO-treated rats were successful at discriminating home cage bedding from fresh bedding. Likewise, virtually all vehicle-treated and low-dose chlordane-treated rats performed successfully in this test, and while most of higher-dose chlordane-treated animals did too, there were significant differences among groups (*P* = 0.0496, one-way ANOVA) (Fig. S11a).

**Fig. 6:**
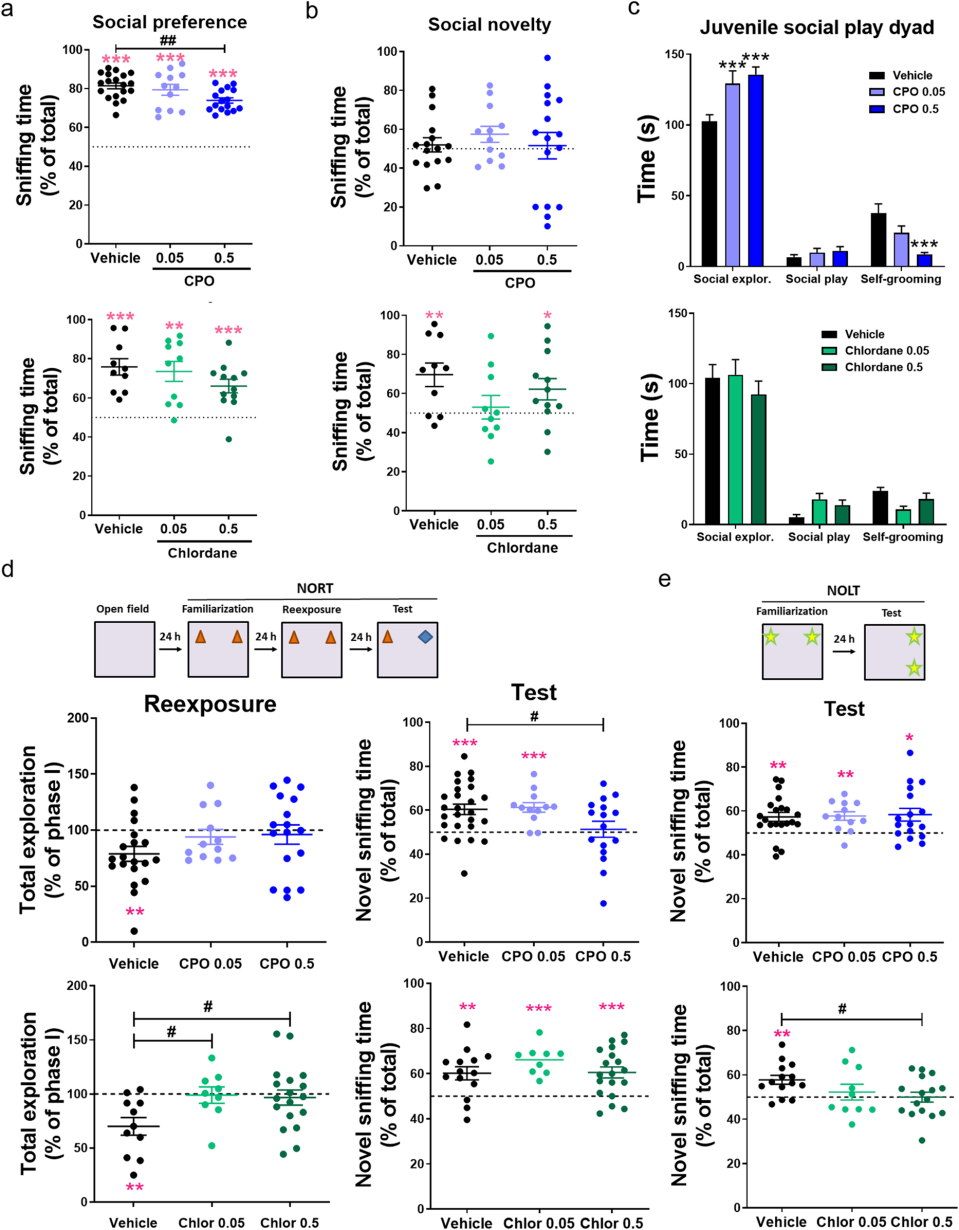
Perinatal exposure to pesticides causes social and cognitive deficits. **a** Percentage of time spent sniffing the unfamiliar rat vs. total time sniffing in the first phase of SPSN. **P < 0.01, **P < 0.001 vs. chance value = 50, one-sample *t*-test; ^**##**^*P* < 0.01, one-way ANOVA + Dunnett’s post-test. **b** Percentage of time spent sniffing the second unfamiliar rat vs. total time sniffing in the second phase of SPSN. *P < 0.05 vs. chance value = 50, one-sample *t*-test; ^**#**^*P* < 0.05, one-way ANOVA + Dunnett’s post-test. **c** Total time of activity in the juvenile social dyad. Mean ± SEM, *n* = 10–20, *P < 0.05, **P < 0.01, ***P < 0.001 vs. vehicle, two-way ANOVA + Dunnett’s post-test. **d** Re-exposure; percentage of time exploring the objects during the re-exposure phase as compared to phase I (familiarization) of NORT. Test; percentage of time exploring the novel object vs. total object exploration time. **P < 0.01, *** *P* < 0.001 vs. reference value = 100 (re-exposure) or 50 (test), one-sample *t*-test; ^**#**^*P* < 0.05, one-way ANOVA + Dunnett’s post-test. **e** Percentage of time exploring the novel location vs total object exploration time during the test phase of NOLT. *P < 0.05, **P < 0.01 vs. chance value = 50, one-sample *t*-test; ^**#**^*P* < 0.05, one-way ANOVA + Dunnett’s post-test

Sociability and preference for social novelty test was later used to study social approach and social memory in two consecutive phases. During the first phase, rats are given the choice to interact with an unfamiliar rat or with an unanimated object. In all groups, rats displayed a preference to interact with the unfamiliar rat versus the object (Fig. 6a). However, this preference was significantly reduced in CPO-treated rats as compared to vehicle-treated rats, indicating decreased social behavior (Fig. 6a, upper plot). A similar trend was found for chlordane-treated rats but the effect did not reach statistical significance (Fig. 6a, lower plot). During the second phase, low-dose chlordane-treated rats did not discriminate the unfamiliar rat from the familiar one as opposed to vehicle-treated rats (Fig. 6b, lower plot). Interestingly, this deficit was mostly observed in males, while no major differences were observed in chlordane-treated females (Fig. S11b). In the case of CPO, all groups showed little social discrimination (Fig. 6b, upper plot).

Sociability was further studied with the juvenile social play dyad, where social exploration and direct play interaction were closely monitored and quantified according to a detailed categorization described in previous work (Ku et al. 2016). Surprisingly, CPO-treated rats spent significantly more time exploring (which includes social sniffing, following, and allogrooming) with the unfamiliar rat. In contrast, no differences were found in time spent playing (including chasing, pouncing, nape attacking, and pinning) although they spent significantly less time self-grooming (Fig. 6c, upper plot). On the other hand, perinatal exposure to chlordane did not significantly affect social investigation or self-grooming although there was a trend for increased social play behavior at both doses (Fig. 6c, lower plot).

Learning and memory were assessed using tests for novel object recognition (NORT) and novel object location (NOLT). These two memory tasks have been associated with hippocampal function, and specifically with hippocampal LTD in vivo (Goh and Manahan-Vaughan 2013a, 2013b). Initially, animals were exposed to two identical objects in an open field arena. During this first phase, all treatment groups spent a similar amount of time exploring the objects (Fig. S12a). Subsequently, the animals were re-exposed to the same (now familiar) objects 24 h later. Consistent with the literature (Goh and Manahan-Vaughan 2013a, 2013b; Zhu et al. 2018), vehicle-treated rats showed reduced object exploration during the re-exposure phase (Fig. 6d, re-exposure, black symbols). In contrast, CPO- and chlordane-treated rats did not reduce their exploration time as compared to the first phase (Fig. 6d, re-exposure, blue and green symbols), indicating that perinatal exposure to these pesticides interfere with object familiarization.

In the third phase of NORT, one of the familiar objects was replaced by a novel object. Vehicle- and low-dose CPO-treated rats spent significantly more time exploring the novel object compared to the familiar one. In contrast, high-dose CPO-treated rats did not discriminate between the two objects (Fig. 6d, test, blue symbols). Conversely, chlordane exposure did not affect memory performance in the NORT (Fig. 6d, test, green symbols). Finally, we used NOLT as a test for spatial memory, where animals were first familiarized with two identical objects and 24 h later they are returned to the same arena where one of the objects has been displaced to a new location. In this case, CPO-treated rats were able to discriminate novel from familiar location, whereas chlordane-treated animals had a dose-dependent impairment in spatial memory as compared to the vehicle group (Fig. 6e). As a control, time spent in the empty corners (whether old familiar or neutral where no objects were ever placed) did not change across the different groups (Fig. S12b).

### Perinatal exposure to pesticides reprograms developmental signaling networks

**Fig. 7:**
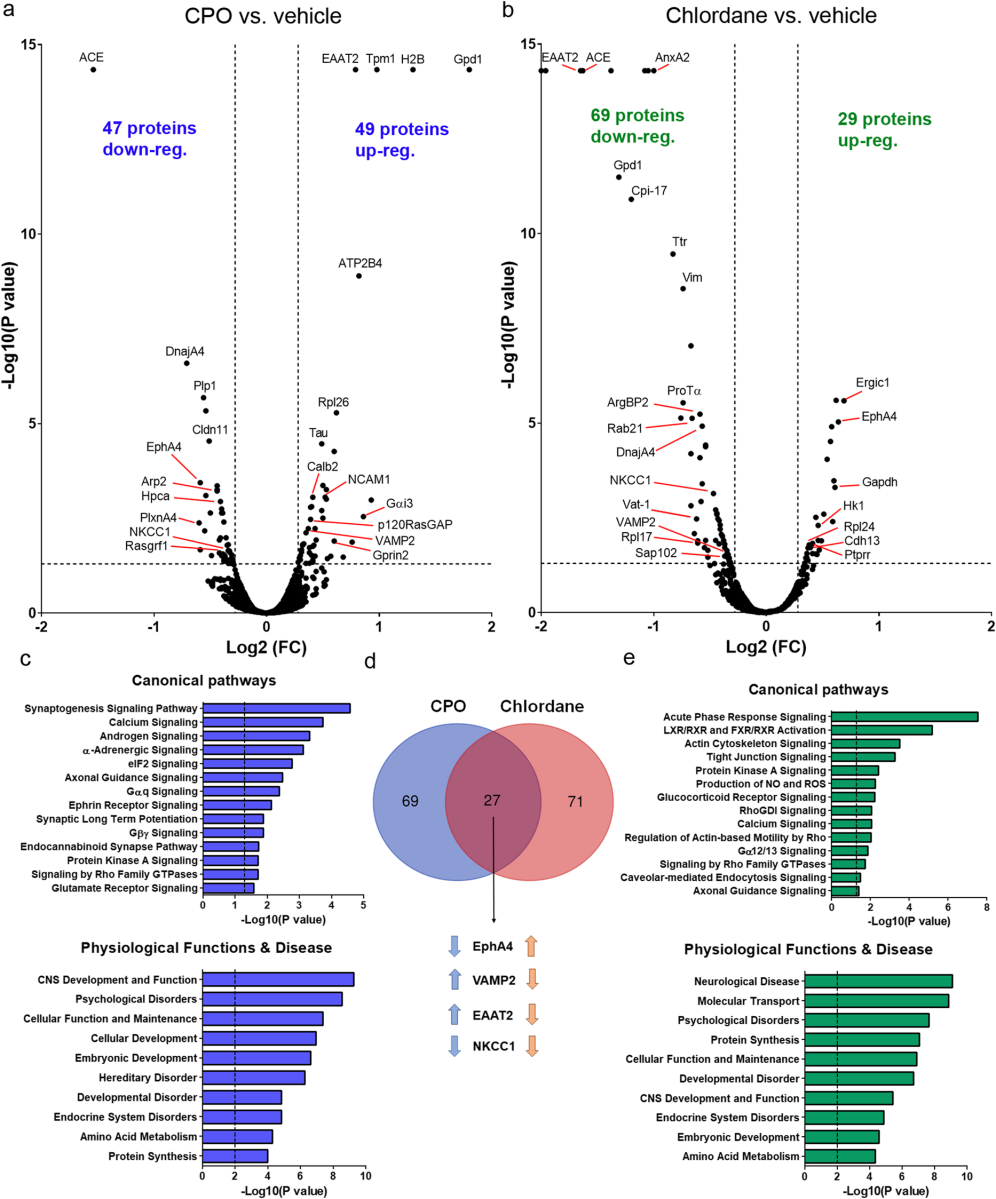
Perinatal exposure to pesticides reprograms developmental signaling networks. **a**, **b** Volcano plots of all the proteins detected in the hippocampus of CPO or chlordane-treated rats (*n* = 4, for all groups) by proteomic analysis as a function of their fold change (FC) and adjusted *P* value as compared to vehicle-treated rats (*n* = 4). Cut-off values were |Log_2_ FC|> 0.28 and *P* < 0.05. Name of relevant, significantly regulated proteins have been highlighted in the plots. **c**, **e** Canonical signaling pathways and physiological functions and disease into which significantly regulated proteins were categorized by Ingenuity pathways analysis. Cut-off values were *P* < 0.05 and *P* < 0.01 for canonical pathways and physiological functions and disease, respectively. **d** Venn diagram of the number commonly regulated proteins for CPO (blue) and chlordane (red). Examples of these proteins are noted below and arrows indicate whether they were upregulated or downregulated by each pesticide

Lastly, in an attempt to assess the effect of pesticides in global developmental programs, we performed proteomic analysis in hippocampal tissue from adult rats exposed to pesticides from gestation through adulthood. In this manner, we aimed at obtaining a more comprehensive insight into the molecular mechanisms and signaling pathways involved in the synaptic and behavioral phenotypes described above. Perinatal exposure to either CPO or chlordane (0.5 mg/kg/day) altered the expression of nearly 100 proteins each (Fig. 7a, b), 27 of which were commonly regulated by both pesticides (Fig. 7d). Among the proteins affected, we found an enrichment in proteins regulating synapse development (synaptogenesis and axonal guidance) such as angiotensin-converting enzyme (ACE), actin-related protein 2 (Arp2), Tau protein, ephrin receptor A4 (EphA4), neural cell adhesion molecule 1 (NCAM1), and plexin A4 (PlxnA4) (Chakrabarty et al. 2008; Suda et al. 2011; Yan et al. 2016; Yang et al. 2019), as revealed by functional analysis using ingenuity pathway analysis (Fig. 7c, e). In addition, we found a high number of ribosomal proteins altered (including Rpl-17, Rpl-24, and Rpl-26) regulating protein synthesis, and specifically eIF2 signaling in the case of CPO. Of note, CPO exposure significantly affected the expression of several proteins related to mGluR signaling (Gαq and Gβγ signaling) such as calmodulin (CaM), Gαi3, and inositol 1,4,5-trisphosphate receptor type 1, as well as proteins regulating the MAPK/ERK pathway like RasGRF1 and p120RasGAP. Ingenuity pathway analysis for the two pesticides also revealed an enrichment of proteins regulating not only embryonic and brain development but also proteins involved in developmental and psychological disorders (Fig. 7c, e). Indeed, 17 out of 27 of the common proteins (regulated by both pesticides) belong to at least one of these categories. Among them, in addition to the abovementioned EphA4, we found the neuronal vesicle-associated membrane protein 2 (VAMP2) and the astroglial excitatory amino acid transporter 2 (EAAT2), which were oppositely regulated by the pesticides, as well as the Na–K-Cl cotransporter 1 (NKCC1), which was downregulated by both CPO and chlordane (Fig. 7d). Western blot analysis was further utilized to complement the proteomic data for some of these proteins in hippocampal lysates from adult male and female rats following perinatal exposure to pesticides. According to our previous findings, NKCC1 protein levels were similarly decreased both in chlordane- and CPO-treated rats as compared to the vehicle group, although the effects were only observed in males but not in females (Fig. S8c). Likewise, the reduction in EAAT2 and VAMP2 levels induced by chlordane exposure were evidenced in males only, whereas CPO had either no effect (in the case of EAAT2) or a trend towards higher VAMP2 levels in females but not in males (Fig. S8d, e). In contrast, EphA levels were significantly enhanced by CPO (but not by chlordane) exposure (Fig. S8d); this could be due to the fact that the antibody used was not specific for EphA4 but it also recognizes the A3 and A5 isoforms. Lastly, the levels of Arp2, CaM, RasGRF1, and Tau were not significantly affected by the pesticides (Fig. S8c,e,f).

## Discussion

The present study provides compelling evidence that developmental exposure to environmentally relevant concentrations of organochlorine and organophosphate pesticides cause molecular, synaptic, and behavioral deficits that resemble those observed in genetic animal models of neurodevelopmental disorders. Such phenotypes include chronic alterations in MAPK/ERK signaling, impaired synaptic plasticity, developmental motor delay, altered social behavior, and cognitive impairment (Borrie et al. 2017; D’Antoni et al. 2014). Furthermore, our findings provide a novel mechanism of synaptic dysfunction shared by several classes of pollutants, namely impaired hippocampal mGluR-LTD, that could link molecular alterations in protein signaling with cognitive and behavioral deficits thereby filling a long-standing gap in the field.

Albeit there has been an open debate on whether CPF/CPO exposure is or not associated with ASD and ADHD (De Cock et al. 2012; Shelton et al. 2012; Williams and DeSesso 2014), more recent animal and human studies show positive correlations between these factors (Von Ehrenstein et al. 2019; De Felice et al. 2015; Gunier et al. 2017; Lan et al. 2017; Roberts et al. 2019; Shelton et al. 2014). This pesticide has been reported to affect motor function and locomotor activity in rodents following prenatal and early postnatal exposure (De Felice et al. 2015; Gómez-Giménez et al. 2018; Laviola et al. 2006; Venerosi et al. 2009), but no such effects were observed when exposed during adolescence or adulthood (Dam et al. 2000; López-Granero et al. 2016; Perez-Fernandez et al. 2020; Savy et al. 2015). In line with these findings, we observed delayed righting reflex test in rats exposed to CPO throughout brain development. In addition, here, we show previously unreported motor defects such as hind limb clasping in these rats. As for locomotor activity, we found that chlordane increased it while CPO had a dose-dependent effect, with higher locomotion at lower doses, a result in agreement with the literature (Gómez-Giménez et al. 2018). The hyperlocomotive effects of organochlorine pesticides have been directly associated with their acute inhibitory action on GABA transmission (Jamaluddin and Poddar, 2001), including those of chlordane (Al-Hachim and Al-Baker 1973; Cassidy et al. 1994). Interestingly, we found that chronic exposure to low concentrations of CPO increased basal GABA_A_R-mediated synaptic responses. However, whether modulation of GABA neurotransmission could underlie the effects of the pesticides on locomotor activity still requires further investigation.

It is becoming clear that an imbalance between excitatory and inhibitory neurotransmission could have an enormous impact on brain development, and it is now considered a hallmark of ASD and associated disorders (Bozzi et al. 2018). Our proteomic analysis revealed bidirectional changes in the astrocytic glutamate transporter EAAT2 induced by the pesticides that could contribute to the unbalance between GABA and glutamate neurotransmitter systems and potentially affect mGluR activity (Huang et al. 2004). These alterations were accompanied by opposite regulation of the astroglial GFAP and microglial Iba1 markers by chlordane and CPO. Considering the importance of immune cells and neuroinflammation in the maintenance of synaptic structure and function during brain development (Faust et al. 2021), future studies should focus on the effects of pesticides in glial cells, particularly in the context of neurodevelopmental disorders. In addition, both pesticides downregulated the expression of NKCC1, which plays a critical role in the switch of GABAergic transmission from excitatory to inhibitory during postnatal development. In this regard, both an early or delayed GABA shift by manipulating NKCC1 expression or activity may not only persistently disturb the GABA/glutamate balance but also cause motor and social behavior disturbances later in adulthood (Peerboom and Wierenga 2021).

Controversies about the link between CPF exposure and ASD have arisen from conflicting results in animal studies regarding the effects of CPF on social and stereotypic behaviors (Williams and DeSesso 2014). We found that perinatal CPO reduced social approach in the social preference test but increased social exploration while reducing self-grooming behavior in the juvenile social play dyad. Despite seemingly paradoxical, our data reproduced previous work where CPF reduced social approach in a social preference test (Lan et al. 2017), where the unfamiliar rodent is inside a wired caged, while increase it in a direct social interaction task (Ricceri et al. 2003) where the animals can freely interact. These findings could be interpreted as enhanced arousal to social cues in pesticide-treated rats, a behavior that may be frustrated in the social preference test where the animals are not able to freely interact and hence rapidly lose interest. In any event, our results may reconcile such controversies in the literature and help to better understand the association between CPF exposure and social behavior. Also, our results in the open field test suggest that perinatal CPO may increase anxiety-like behavior in rats, and are consistent with other studies that reported enhanced thigmotaxis and anxiety in rats exposed to CPF/CPO (Braquenier et al. 2010; López-Granero et al. 2016; Mullen et al. 2013; Venerosi et al. 2012). Therefore, taken all together, our findings support the notion that perinatal exposure to CPO reproduce behavioral alterations typical of neurodevelopmental disorders, including altered social behavior, impaired learning and memory, increased anxiety, and delayed motor development.

Our results also revealed that chronic exposure to several classes of pesticides enhance MAPK/ERK signaling at low, environmentally relevant concentrations. Considering the pleiotropic functions of MAPK during neurodevelopment, chronic alterations of this pathway by environmental contaminants could be expected to profoundly affect many different aspects of brain development, either directly by modulating gene expression or by interfering with endogenous neurotrophic factors and/or hormones, possibly leading to increased risk and/or severity of neurodevelopmental disorders (Borrie et al. 2017; Bustelo et al. 2018; Carter and Blizard 2016; Courchesne et al. 2020; Schantz and Widholm 2001). In this regard, many of the environmental contaminants included in our study have been shown to act as endocrine disruptors in the brain (Briz et al. 2011; Li et al. 2019). In cases like organochlorine pesticides, the doses at which pollutants interfere with hormone receptor/signaling (including ERK activation) are 1–2 orders of magnitude higher than the one used here. Nevertheless, they could also affect hormone receptor expression and/or hormone levels at more relevant nM concentrations (Briz et al. 2011; Li et al. 2019), and hence indirectly affect hormone-regulated neurodevelopment. Also, the ability of some pesticides to cause persistent epigenetic defects that are transmitted to the progeny should be also considered as a potential mechanism for disrupting brain development (Anway et al. 2005). In any event, we found that perinatal pesticide exposure altered the expression levels of several signaling molecules critical for brain development such as EphA4, Arp2, NCAM1, and PlxnA4. Future studies should investigate the role of these proteins in the deleterious effects of pesticides and other pollutants on neurodevelopment, as some of them have been associated with ASD (Suda et al. 2011; Yan et al. 2016; Yang et al. 2019).

In our hands, the effects of CPO on MAPK activation and mGluR-LTD were observed at concentrations (10–100 nM) and doses (0.5 mg/kg) compatible with acetylcholinesterase inhibition after chronic exposure in organotypic slices and in vivo following early postnatal exposure (Betancourt and Carr 2004; Prendergast et al. 2007), respectively. Thus, a plausible mechanism for CPO-induced mGluR-LTD impairment could be via direct or indirect modulation of muscarinic acetylcholine receptors (Huff et al. 1994; Liu et al. 2002). In fact, we observed that chronic blockade of muscarinic receptors with atropine interfered with mGluR-LTD, a result consistent with the interplay between mGluRs and muscarinic receptors in mediating synaptic depression (Ghoshal et al. 2017; Kamsler et al. 2010; Volk et al. 2007). The convergent signaling from these two types of receptors has been shown to increase inhibitory synaptic transmission (Ghoshal et al. 2017). Interestingly, our results indicate that CPO produces an increase in GABA_A_ receptor synaptic currents, and these effects were mimicked (in a non-additive manner) by the muscarinic receptor antagonist atropine. Furthermore, our proteomic analyses showed that CPO regulates the expression of proteins involved in Gαq and Gβγ signaling, which are downstream of both mGluR1/5 and muscarinic receptors. Therefore, our findings strongly suggest that interference with muscarinic receptor signaling, possibly via GABAergic transmission, is responsible for the impairment of mGluR-LTD induced by CPO.

In the case of chlordane, using subthreshold doses of a MAPK inhibitor we were able to prove that chronic elevation of this signaling cascade is responsible for the effect of this pollutant on synaptic function. This interpretation is in good agreement with genetic mouse models in which chronic enhancement of MAPK/ERK activity in the hippocampus is responsible for mGluR-LTD impairment (Chévere-Torres et al. 2012; Schreiber et al. 2017). However, it is worth noting that even though these two contaminants (chlordane and CPO) enhanced ERK signaling, only in the case of chlordane mGluR-LTD impairment was MAPK dependent. These mechanistic differences may underlie the differing cognitive effects of these pollutants on some behavioral tasks (i.e., NORT vs NOLT). There was one memory test, however, that was similarly affected by the two pesticides, that is object familiarization (the re-exposure phase of NORT). Interestingly, this specific memory task has directly been associated with in vivo LTD in the hippocampus (Goh and Manahan-Vaughan 2013a, 2013b), and is also impaired in mice with deficits in mGluR-LTD (Zhu et al. 2018). Thus, our results strengthen the association between hippocampal mGluR-LTD and object familiarization, irrespective of the intracellular signaling pathway leading to synaptic dysfunction. Given that mGluR-LTD is known to be expressed in other brain regions, including the neocortex, midbrain, and cerebellum (Lüscher and Huber 2010), future work should investigate whether impaired mGluR-LTD is a general feature of synaptic disruption caused by pesticides in the brain.

In summary, the present study identifies a common mechanism of synaptic dysfunction shared by different classes of pesticides, namely modulation of MAPK/ERK signaling and impaired mGluR-LTD in the hippocampus, which may represent critical pathophysiological substrates linking exposure to environmental contaminants and increased risk for neurodevelopmental disorders. In the light of recent regulatory policies to discontinue the use and production of CPF in the EU (SANTE/11938/2019) and recommendations urging to take similar actions in the US (Hertz-Picciotto et al. 2018), our findings provide mechanistic insights for the association between exposure to CPF and neurodevelopmental disorders, and strengthen the scientific evidence to support these regulatory measures worldwide.

### Supplementary Information

Below is the link to the electronic supplementary material.

Supplementary file1 (DOCX 10.1 MB)

Supplementary file2 (XLSX 469 KB)

## Data Availability

All data will be made available upon request.
