# Supplementary material for: Perinatal exposure to pesticides alters synaptic plasticity signaling and induces behavioral deficits associated with neurodevelopmental disorders
Source: Cell Biol Toxicol. 2022 Feb 8;39(5):2089–111. doi: 10.1007/s10565-022-09697-2 (PMC10547633; doi:10.1007/s10565-022-09697-2)
Supplement: Supplementary file 1 — Supplementary file1 (DOCX 10.1 MB) [file 10565_2022_9697_MOESM1_ESM.docx]

**SUPPLEMENTARY INFORMATION**

**Perinatal exposure to pesticides alters synaptic plasticity signaling and induces behavioral deficits associated with neurodevelopmental disorders**

Cell Biology and Toxicology

Esperanza López-Merino^1^, María I. Cuartero^2^, José A. Esteban^1,^* and Víctor Briz^1,^*

^1^Centro de Biología Molecular Severo Ochoa (CSIC-UAM), Madrid, Spain.

^2^ Neurovascular Pathophysiology Group, Centro Nacional de Investigaciones Cardiovasculares Carlos III (CNIC), Madrid, Spain.

* To whom correspondence should be addressed at:

E-mail: [victor.briz@cbm.csic.es](mailto:victor.briz@cbm.csic.es)

E-mail: [jaesteban@cbm.csic.es](mailto:jaesteban@cbm.csic.es)

**SUPPLEMENTARY FIGURES**

**
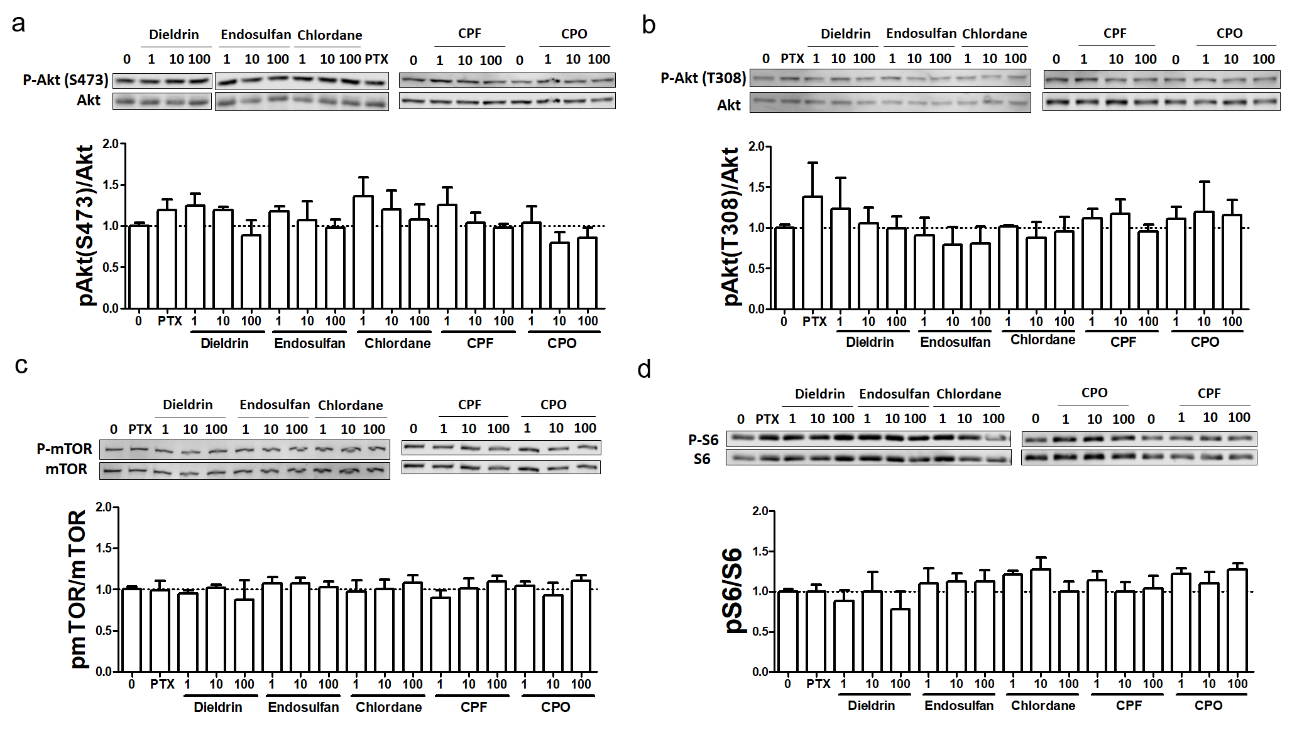
Figure S1. Chronic effects of pesticides on Akt/mTOR/S6 signaling.** Western blots of P-Akt vs. total Akt (**a,b**), P-mTOR vs. total mTOR (**c**) and P-S6 vs. total S6 (**d**) in organotypic hippocampal slices exposed chronically to different concentrations of pesticides. Mean ± SEM, n = 3-6.

**
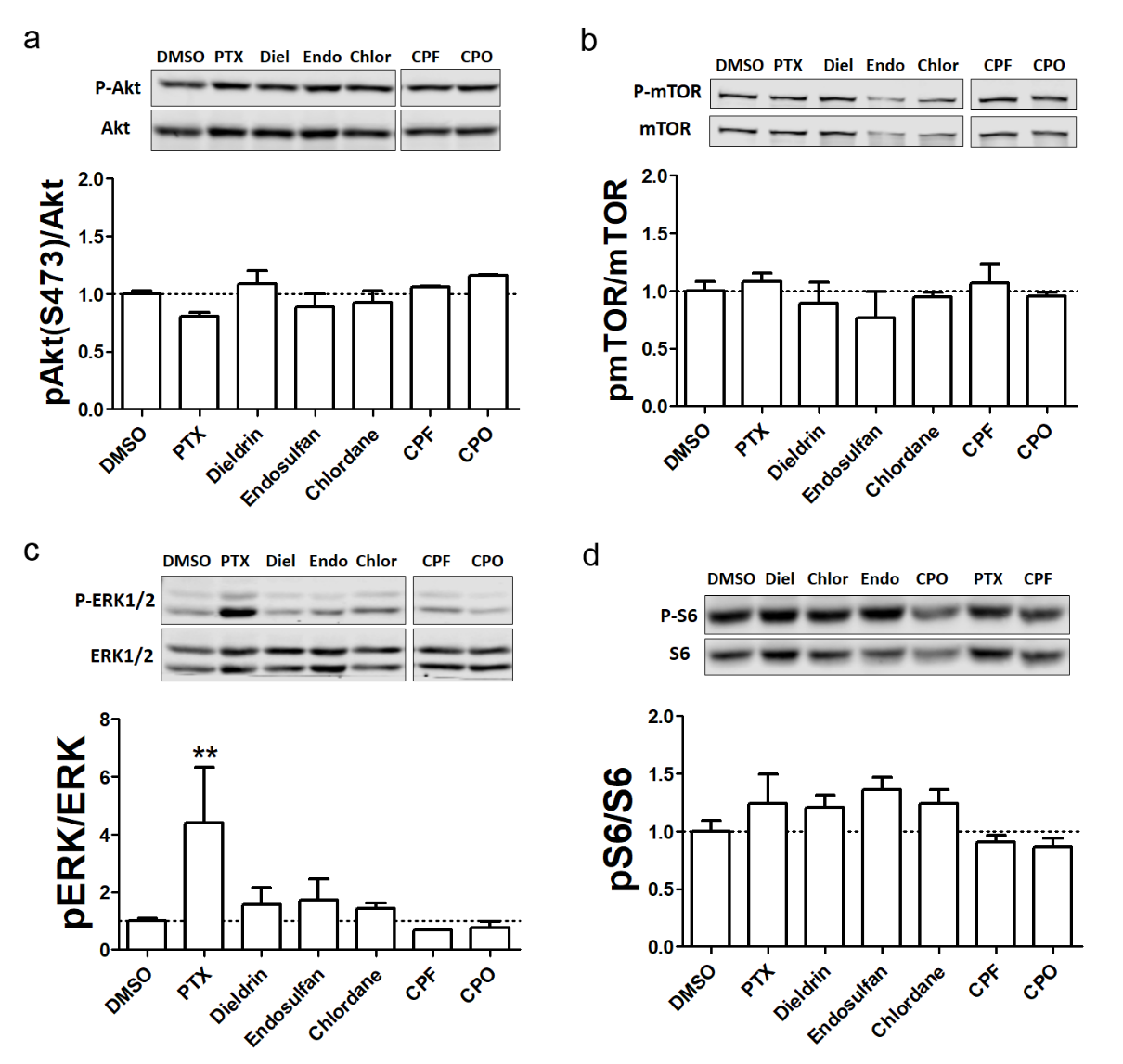
Figure S2. Acute effects of pesticides on Akt/mTOR/S6 and MAPK/ERK signaling.** Western blots of P-Akt vs. total Akt (**a**), P-mTOR vs. total mTOR (**b**), P-ERK1/2 vs. total ERK1/2 (**c**) and P-S6 vs. total S6 (**d**) in organotypic hippocampal slices exposed acutely (for 2h) to pesticides (at 100 nM). Mean ± SEM, n = 3-5, **P < 0.01 vs. DMSO, one-way ANOVA + Dunnett’s post-test.

**
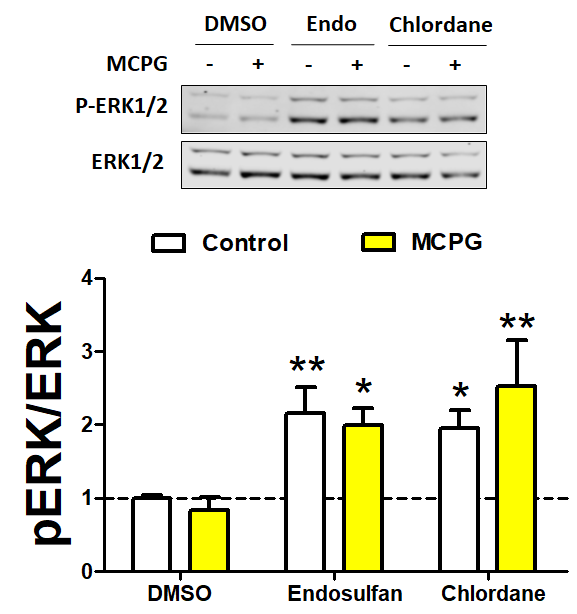
**

**Figure S3. Effect of mGluR1/5 inhibition on pesticide-induced MAPK/ERK1/2 activation.** Western blots of P-ERK1/2 vs. total ERK1/2 in organotypic hippocampal slices exposed chronically to contaminants in the absence or presence of 500 µM MCPG. Mean ± SEM, n = 3-7, *P < 0.05, **P < 0.01 vs. DMSO, two-way ANOVA + Bonferroni’s post-test.

**
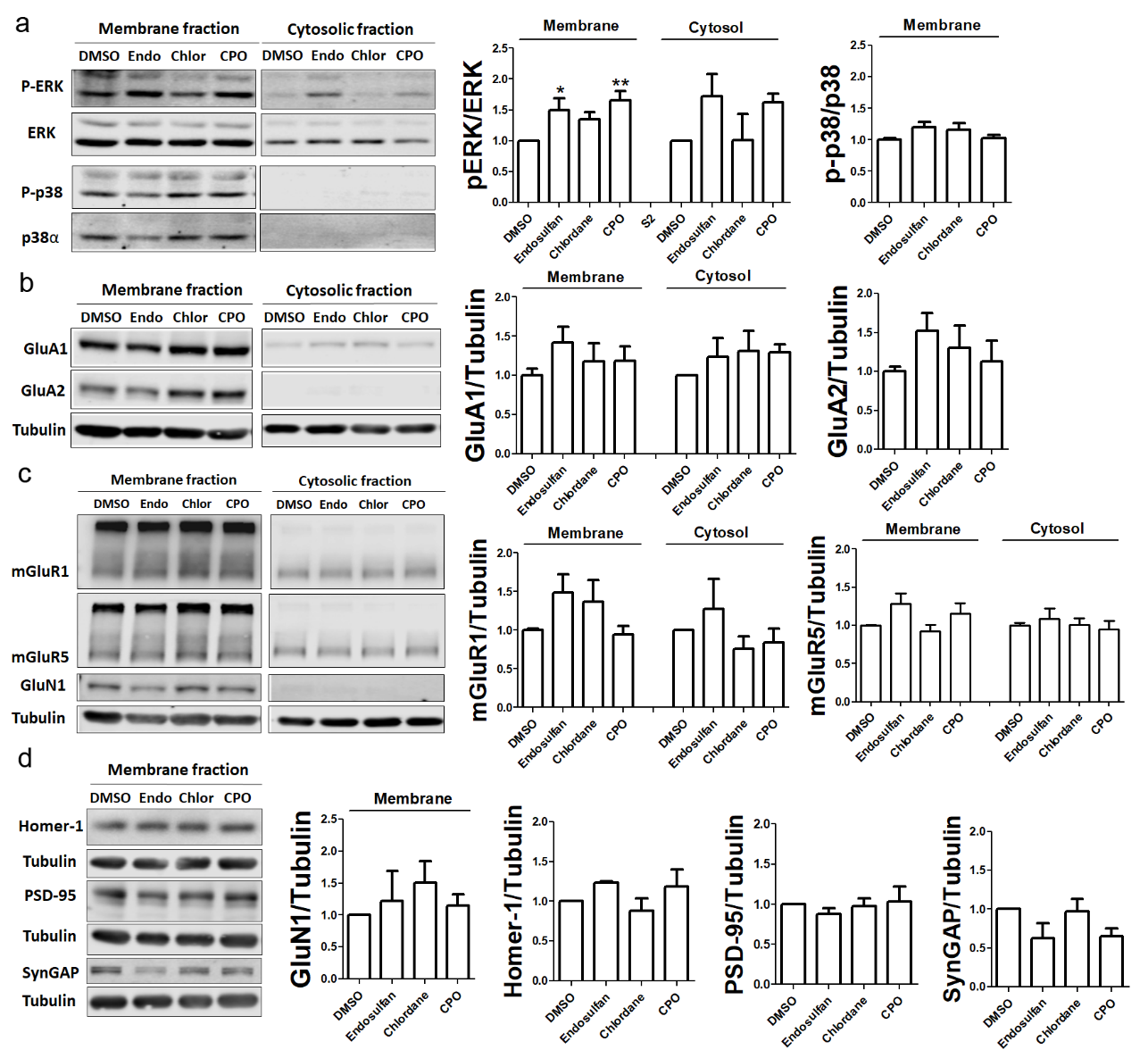
Figure S4. Chronic effects of pesticides on the levels of glutamate receptor and associated synaptic proteins in membrane preparations.** Western blots of P-ERK1/2 vs. total ERK1/2, P-p38 vs total p38α (**a**) and different glutamate receptor subunits (**b,c**) and synaptic proteins (**d**) in membrane and cytosolic fractions from organotypic hippocampal slices exposed chronically to pesticides. Mean ± SEM, n = 3-10, *P < 0.05, **P < 0.01 vs. DMSO, one-way ANOVA + Dunnett’s post-test.

**
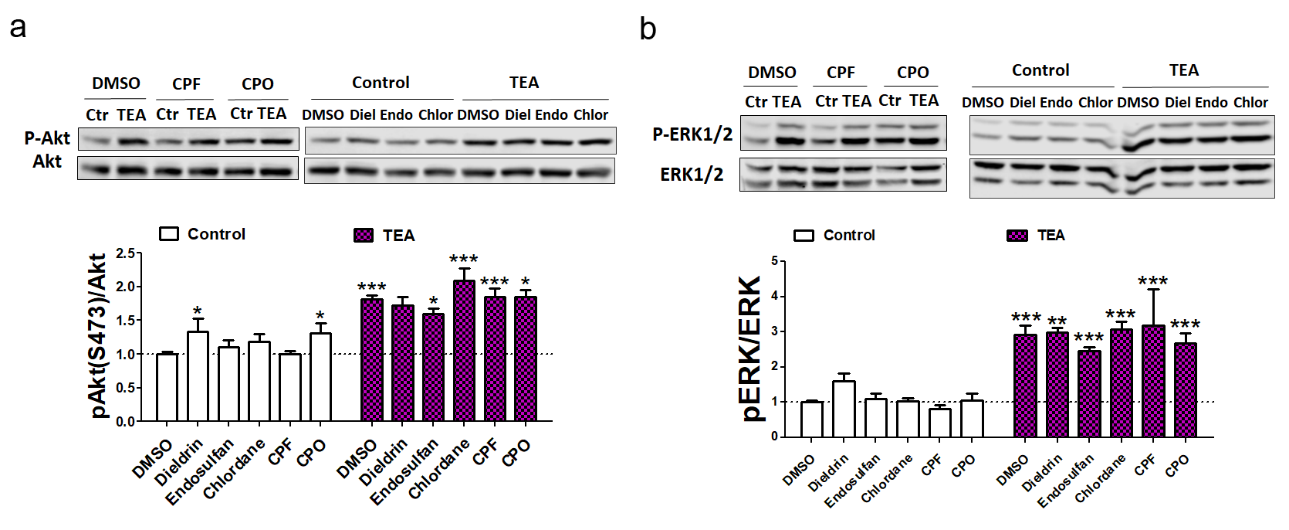
Figure S5. Effects of environmental contaminants on TEA-induced kinase activation.** Western blots of P-Akt vs. total Akt (**a**) and P-ERK1/2 vs. total ERK1/2 (**b**) after TEA treatment in slices exposed chronically to pesticides. Mean ± SEM, n = 3-8, *P < 0.05, **P < 0.01, ***P < 0.001 vs. control, two-way ANOVA + Bonferroni’s post-test.

**
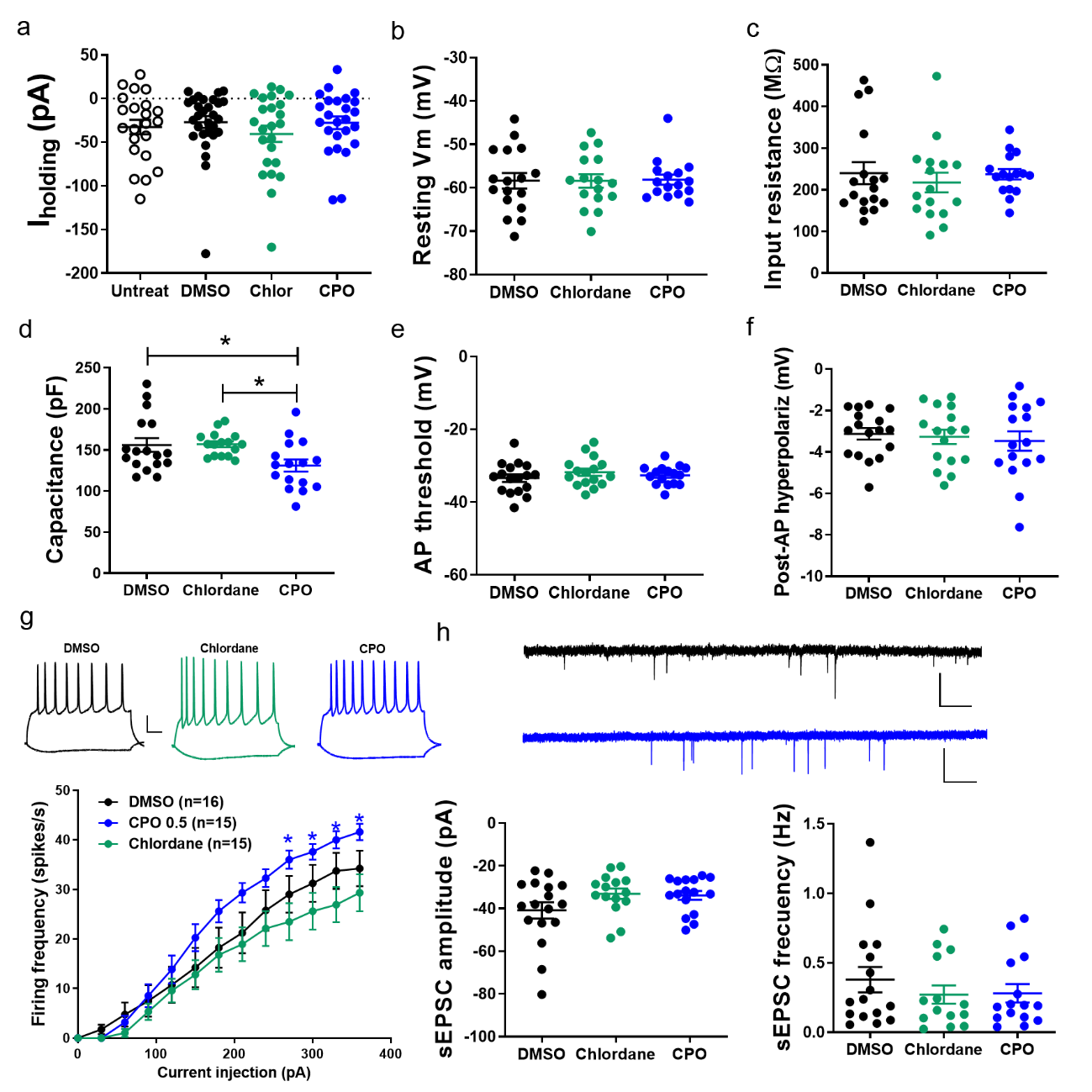
**

**Figure S6. Effects of chlordane and CPO on passive cell membrane properties, excitability and spontaneous activity.** Patch**-**clamp recordings of CA1 pyramidal neurons from organotypic slices exposed chronically to pesticides or DMSO. Non-treated slices (untreat) were used as control. (**a**) Holding currents (I_holding_) of cells clamped at -60 mV during basal transmission recordings. Mean ± SEM, n= 22-29. (**b-f**) Resting membrane potential (Vm), input resistance, capacitance, action potential (AP) threshold and post-AP hyperpolarization of cells during current injection experiments. Mean ± SEM, n= 16-17. (**g**) Representative traces of cells at I=-60 pA and I=360 pA (above) and quantification of firing frequency (below) during current injection experiments. Scale bars = 20 pA/50 ms. (**h**) Representative traces (above) and quantification of spontaneous excitatory postsynaptic currents (sEPSC) amplitude and frequency. Scale bars = 40 pA/2 s.

**
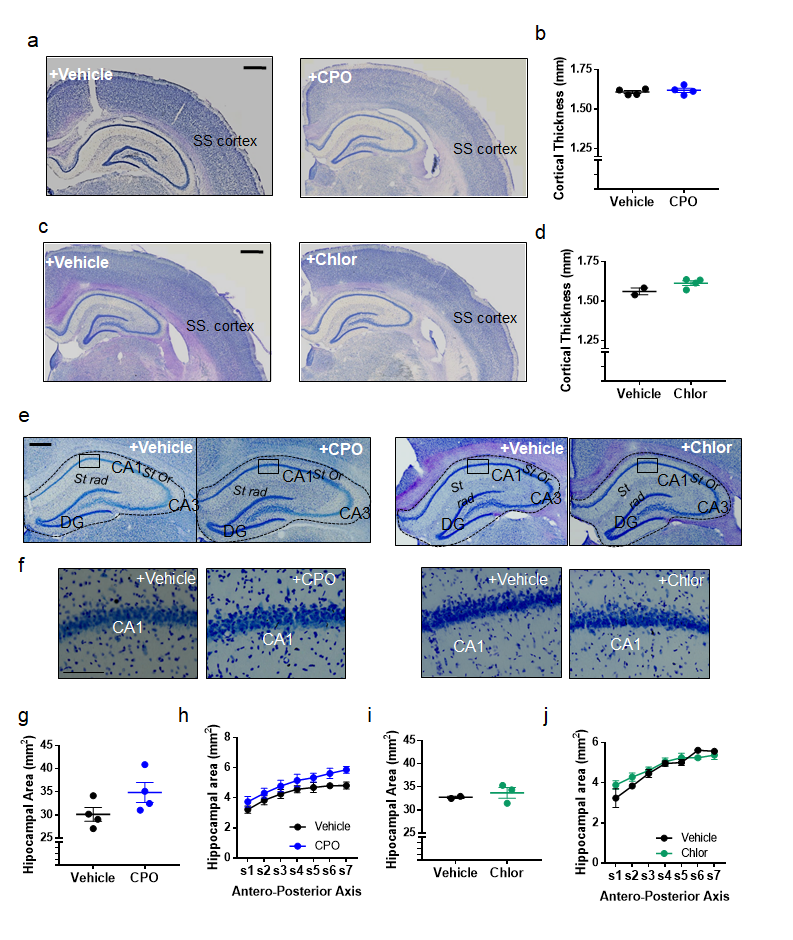
**

**Figure S7. Effects of perinatal exposure to pesticides on cortical and hippocampal size.** (**a,c**) Representative images of Nissl staining in brain sections from vehicle-, chlordane- and CPO-treated rats. (**b,d**) Quantification of the thickness of the somatosensory (SS) cortex. (**e,f**) Representative images of Nissl staining and (**g-j**) quantification of the total hippocampal area along the rostro-caudal axis in vehicle- and pesticide-treated rats. Data are mean ± SEM (n=2-4). Scale bar= 1000µm in **a,c** and 500µm in **e,f**. SS cortex (somatosensory cortex), St.rad (stratum radiatum); St. Or (Stratum Oriens); DG (dentate gyrus).


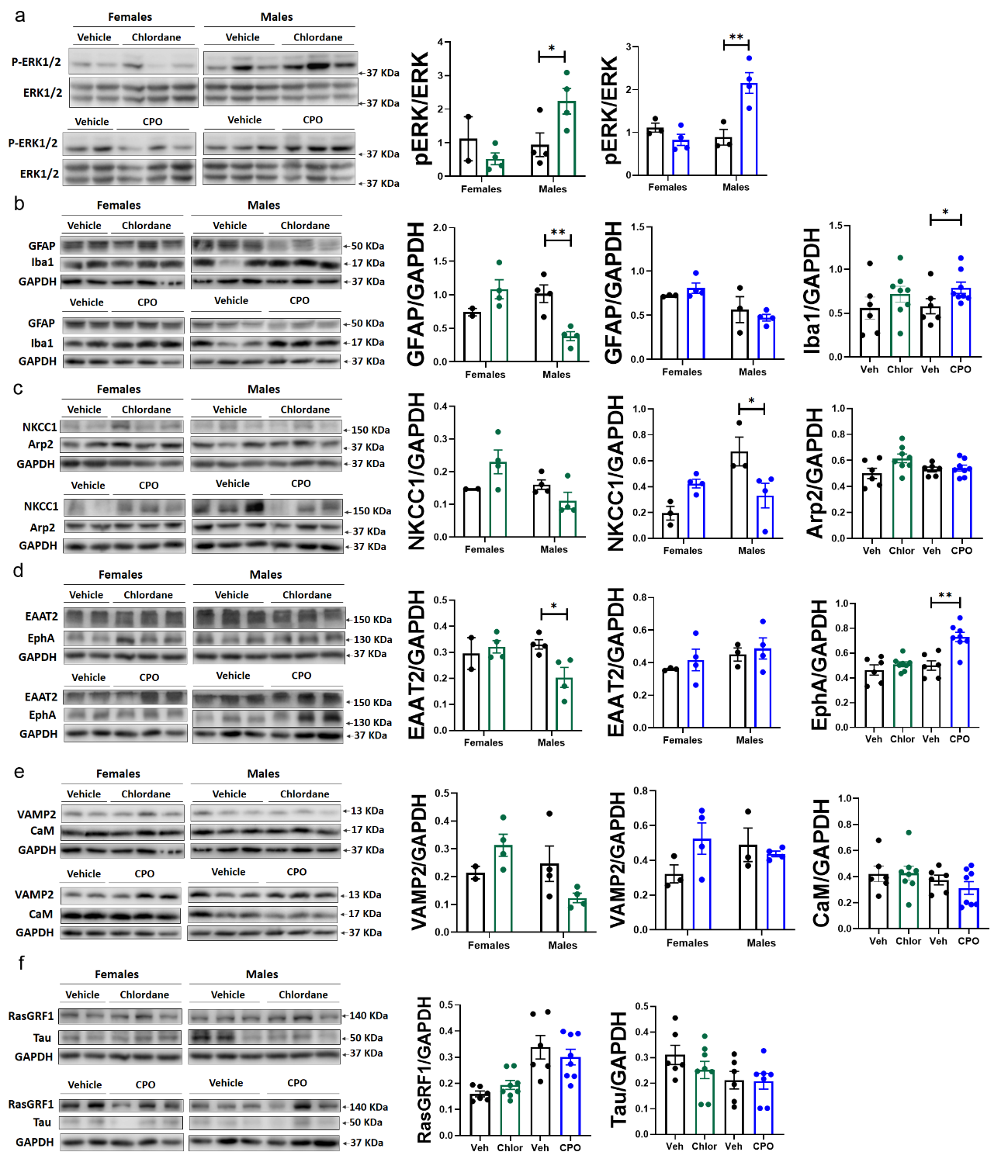


**Figure S8. Hippocampal levels of proteins in rats exposed to pesticides.** Western blots of P-ERK1/2 vs. total ERK1/2 (**a**), glial proteins (**b**) and several proteins identified by proteomics (**c-e**) in hippocampal lysates from adult rats exposed chronically to pesticides. Mean ± SEM, n = 6-8, *P < 0.05, **P < 0.01 vs. vehicle, two-way ANOVA + Bonferroni’s post-test or Mann-Whitney test.

**
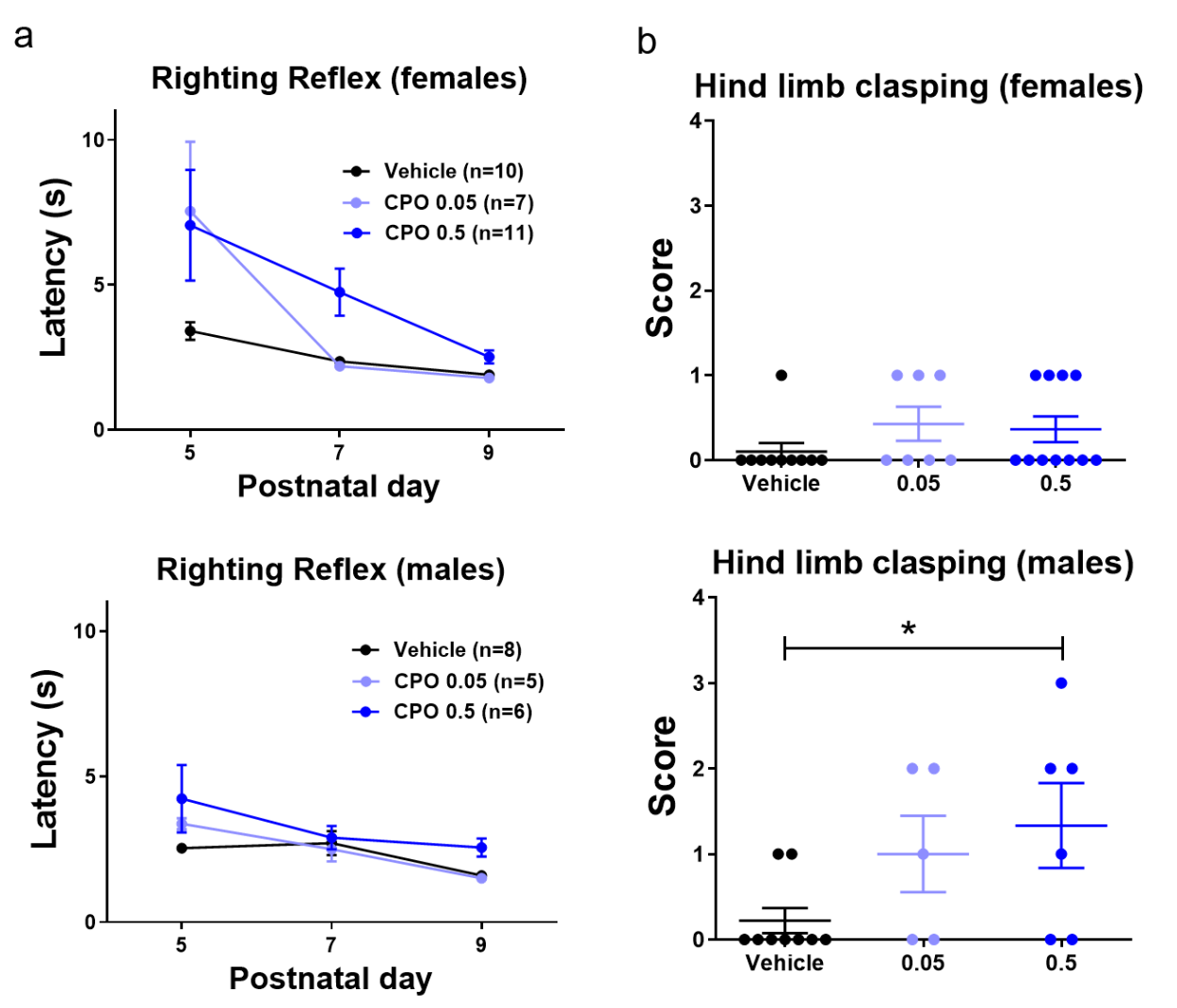
Figure S9. Sex-dependent effects of perinatal exposure to CPO on righting reflex and hind limb clasping.** (**a**) Latency to righting all four limbs during the first days of postnatal period in male vs. female rats. Mean ± SEM, values from 3 consecutive trials were averaged for each rat pup. (**b**) Hind limb clasping score at weaning (PND21). *P < 0.05, one-way ANOVA + Dunnett’s post-test.

**
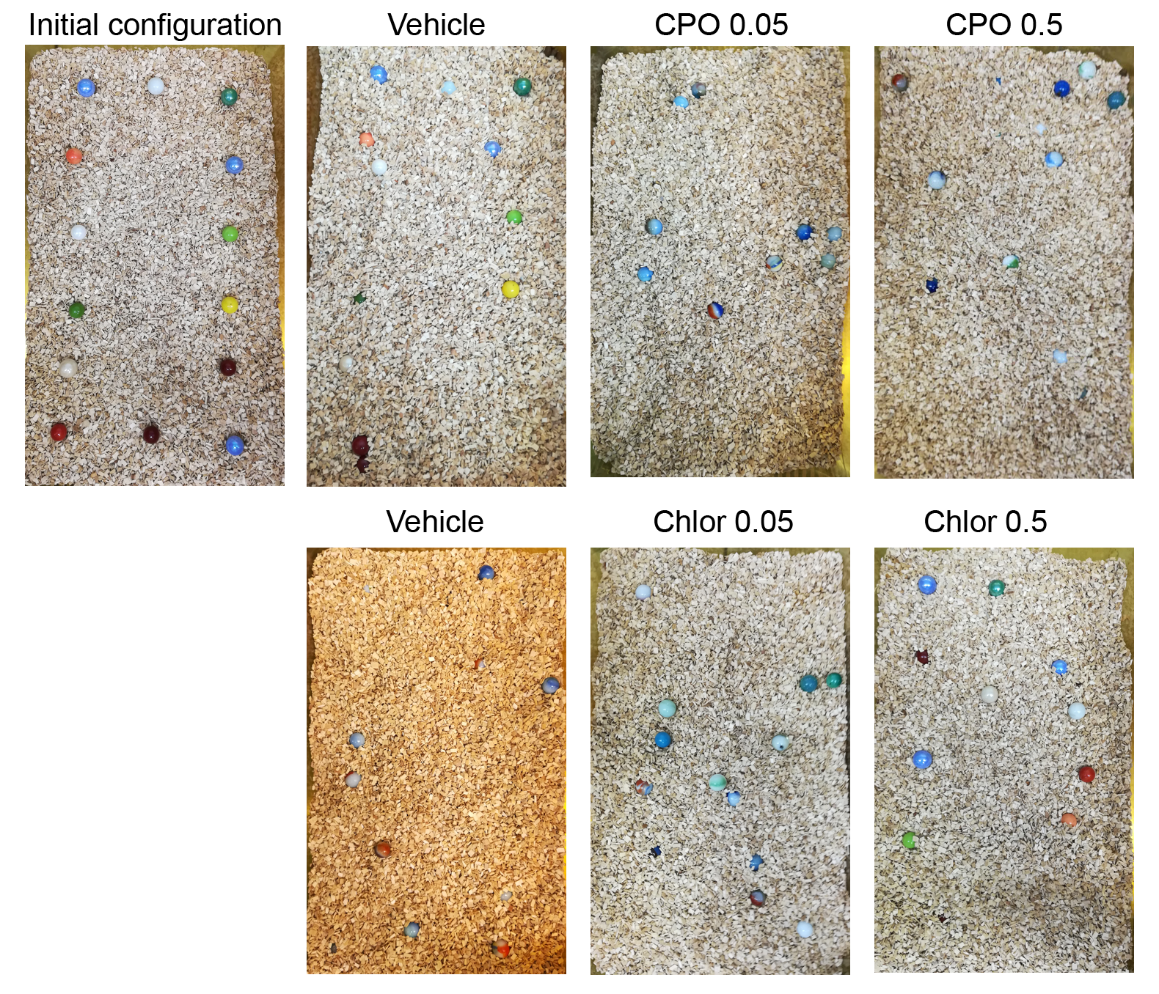
**

**Figure S10. Representative pictures of the marble burying test.** Picture depicting the initial configuration of the marbles in the cage before the test, and representative pictures for the different treatment groups after the test.

**
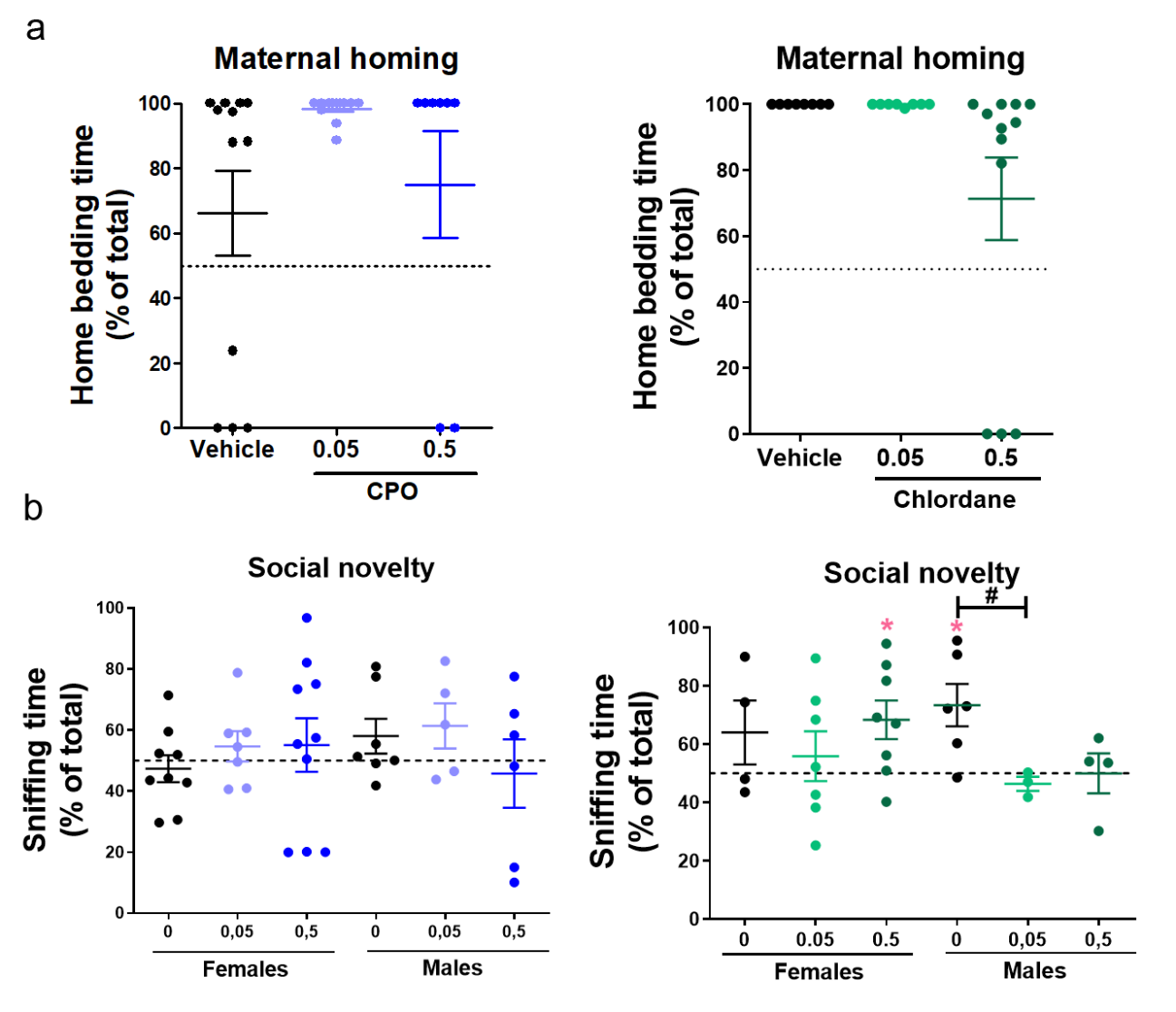
Figure S11. Effects of perinatal exposure to pesticides on maternal homing test and social novelty.** (**a**) Percentage of time spent in home bedding corner vs total time exploring. P = 0.0496, one-way ANOVA (chlordane). (**b**) Percentage of time spent sniffing the second unfamiliar rat vs. total time sniffing in the second phase of SPSN. *P < 0.05 vs. chance value=50,one-sample *t*-test; **^#^**P < 0.05, one-way ANOVA+ Dunnett’s post-test.

**
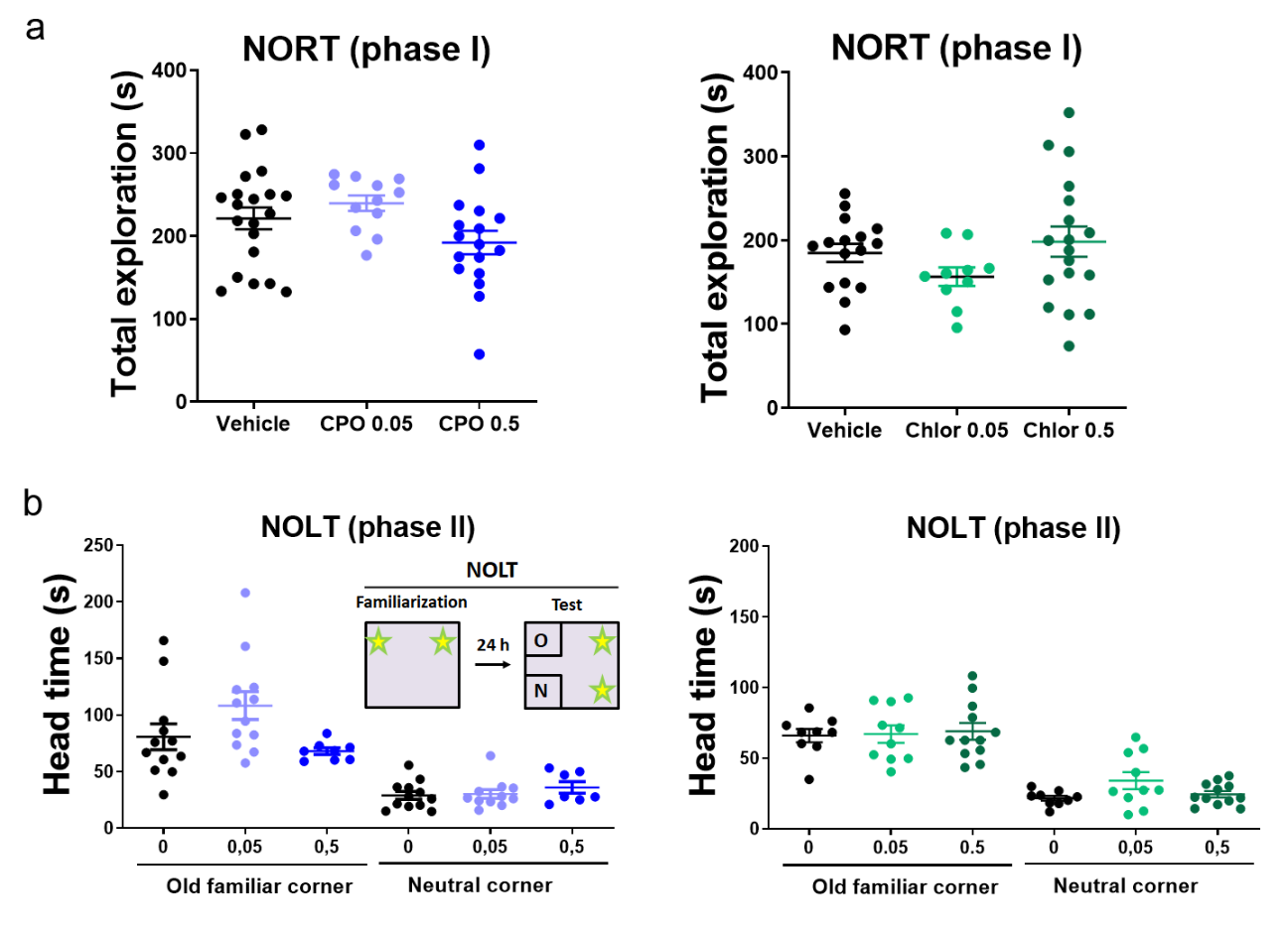
 Figure S12. Effects of perinatal exposure to pesticides on object exploration in different phases of NORT and NOLT.** (**a**) Total time exploring the objects during phase I (familiarization) of NORT. (**b**) Total time exploring the corner where the old familiar object was during phase I of NOLT (corner O in scheme) vs. time exploring the neutral corner (corner N in scheme) during the second phase of NOLT.

**EXTENDED MATERIALS AND METHODS**

**Organotypic hippocampal slices**

Hippocampal slices were dissected from postnatal day (PND) 5-7 rats in ice-cold dissection medium (10 mM D-glucose, 4 mM KCl, 20 mM NaHCO_3_, 233.7 mM sucrose, 5 mM MgCl_2_, 1 mM CaCl_2_) gassed with carbogen (5% CO_2_/95% O_2_). Four hundred μm-thick slices were prepared in sterility using a McIlwain tissue chopper, and individual slices were separated and placed in culture on porous membranes (Merck Millipore) over culture medium (0.8% [w/v] MEM powder, 20% [v/v] horse serum, 1 mM L-glutamine, 1 mM CaCl_2_, 2 mM MgSO_4_, 1 mg/L insulin, 0.0012% [v/v] ascorbic acid, 30 mM HEPES, 13 mM D-glucose, 5.2 mM NaHCO_3_). Slices were maintained in vitro at 35.5°C and 5% CO_2_ for 6-11 days until use, replacing every 2-3 days with fresh medium containing the selected concentrations of pollutants.

**Reagents and treatments**

All contaminants were purchased from Sigma except for chlorpyrifos-oxon (CPO) which was obtained from ChemService. All stock solutions were prepared in DMSO at 1000X; final DMSO concentration in the culture medium was 0.1% in all conditions. In some experiments, slices were chronically or acutely treated with the GABA_A_R antagonist picrotoxin (PTX, 100 µM), the NMDAR antagonist AP5 (50 µM), the mGluR1/5 antagonist MCPG (50 µM), the muscarinic receptor antagonist atropine (0.1 µM) or the MAPK inhibitor PD98059 (0.1-10 µM).

**Electrophysiology**

Whole-cell voltage-clamp recordings were obtained from CA1 pyramidal neurons in organotypic hippocampal slice cultures, evoking synaptic responses via stimulation of Schaffer collateral fibers from CA3 pyramidal neurons. The CA3 region was cut off from the slices to prevent propagation of recurrent CA3 activity. Slices were maintained in an immersion recording chamber that was continuously perfused with aCSF containing 4 mM CaCl_2,_ 4 mM MgCl_2_ and 4 μM 2-chloroadenosine, constantly gassed with 5% CO_2_ / 95% O_2_ (carbogen) and maintained at 29°C. Patch recording pipettes (3–8 MΩ) were filled with a solution containing 115 mM CsMeSO_3_, 20 mM CsCl, 10 mM HEPES, 2.5 mM MgCl_2_, 4 mM Na_2_-ATP, 0.4 mM Na-GTP, 10 mM sodium phosphocreatine, 0.6 mM EGTA, 10 mM lidocaine N-ethyl bromide, pH 7.25 and 290 mOsm, for voltage clamp experiments. For current clamp experiments, an internal solution containing 115 mM K gluconate, 20 mM KCl, 10 mM HEPES, 2 mM MgCl_2_, 4 mM Na_2_-ATP, 0.3 mM Na-GTP was used. Liquid junction potential was corrected using the Pipette Offset feature on the amplifier. Stimulating bipolar electrodes were placed over Schaffer collateral fibers at 200-400 μm from the recorded cells with visual guidance using transmitted light illumination under a microscope equipped with a 60x water immersion objective. Synaptic responses were evoked using single-voltage pulses (200 μs, up to 40 V) and electrophysiological recordings and data acquisition were performed with Multiclamp 700A/B amplifiers, a digitizer and pClamp software (Molecular Devices). Data analysis was performed with custom-made Excel (Microsoft) macros (Brachet et al. 2015).

Synaptic AMPAR-, NMDAR- and GABA_A_R-mediated responses were measured at -60 mV, +40 mV, and 0 mV, respectively, in aCSF supplemented with 100 μM PTX (AMPAR and NMDAR responses) or 100 μM AP5 (GABA_A_R responses). Responses were averaged over 50 trials. Passive cell membrane properties such as resting membrane potential, input resistance and capacitance were measured under current clamp configuration, whereas holding current was measured under voltage clamp configuration (-60 mV). Intrinsic excitability of neurons, including AP threshold, post-AP hyperpolarization and firing frequency were obtained by current injection steps, from -60 pA to 360 pA in steps of +30 pA. Spontaneous excitatory postsynaptic currents (sEPSC) were measured in aCSF for 2-3 min under voltage clamp configuration (-60 mV), and keeping CA3-CA1 connections intact. Miniature inhibitory postsynaptic currents (mIPSC) were measured in aCSF containing 100 μM AP5, 10 μM CNQX and 1 μM tetrodotoxin (TTX) under voltage clamp configuration (-60 mV), and replacing CsMeSO_3_ with CsCl in the internal solution. NMDAR-dependent LTD was induced in CA1 neurons by pairing 1 Hz (300 pulses) presynaptic stimulation of the Schaffer collaterals with -40 mV postsynaptic depolarization. mGluR-LTD was induced by paired pulse low frequency stimulation (PP-LFS, 900 paired pulses, separated by 50 ms at 1 Hz) in the presence of 100 µM AP5 and 100 μM PTX, and omitting 2-chloroadenosine. To induce LTP, a modified aCSF containing 25 mM tetraethylammonium (TEA) was incubated for 10 min under current clamp configuration to allow action potential bursting.

**Western blotting and antibodies**

Ten to 30 µg of protein were subjected to 6-15% sodium docecyl sulfate–polyacrylamide gel electrophoresis, as previously described (Briz and Baudry 2014). Membranes were scanned using an Odyssey infrared imager (LI-COR) or ImageQuant LAS 4000 (GE Healthcare), and protein levels were quantified using Image Studio Lite version 5.2 (LI-COR). For information on the primary and secondary antibodies used see Tables S1 and S2 (below), respectively.

For membrane fractionation experiments, 4-6 cultured slices were pooled together and homogenized in sucrose buffer (0.32M sucrose, 1mM HEPES -pH 7.4-, 1 mM MgCl_2_, 1mM NaHCO_3_, 1mM EDTA) containing protease/phosphatase inhibitor cocktail (Roche), and centrifuged at 1000 g for 10 min. The resulting supernatant was centrifuged 20 min at 11,000 g and the pellet collected as crude synaptosomal fraction or membrane fraction, while the supernatant was kept as cytosolic fraction. Samples were then processed for western blot or stored at -80 ºC until use.

**Puromycin labelling**

Protein synthesis was measured by using puromycin labelling as previously described (Briz et al. 2017), with minor modifications. Briefly, cultured hippocampal slices were pre-incubated with puromycin (5 μg/ml) in aCSF for 30-35 min, and then chemical treatments (cLTP or DHPG) were applied as described earlier in the presence of puromycin. Slices treated with cycloheximide (25 μM) 30 min before adding puromycin were used as negative control and slices not treated with puromycin were used as blank for quantification. After washing out the drugs, slices were homogenized and processed for western blot using an anti-puromycin primary antibody (DHSB, PMY-2A4) and a donkey anti-mouse horseradish peroxidase secondary antibody (Jackson), and developed using a chemiluminiscence system. Ponceau red (1 mg/ml in 1% acetic acid solution) staining of total protein content was used as loading control for normalization.

**Perinatal exposure to pesticides**

Chlordane (technical grade, Sigma) and CPO (Chem Service) stock solutions were prepared in DMSO at 150-200 mg/ml. The precise concentration for each stock was quantified using gas chromatography and mass spectrometry (GC-MS) by the laboratory of Prof. Mariana F. Fernández at University of Granada. Oral exposure to CPO was through drinking water, as it has been shown to better mimic the potential exposure to different classes of contaminants occurring in humans (Bisen-Hersh et al. 2014; Lin et al. 2014). The doses of CPO (0.05-0.5 mg/Kg/day) selected were at least 10 times lower than those reported to have behavioral effects (Laviola et al. 2006; Mullen et al. 2013), the highest one being sufficient to inhibit acetylcholine esterase (ACE) in the forebrain (Betancourt and Carr 2004). The final concentration of CPO in the water bottle ranged between 2-6 µg/ml as it was adjusted to animal weight and daily water consumption in order to keep the dosage constant. At this range of concentrations, the amount of DMSO was always less than 0.001%. New polycarbonate water bottles were used, as they have been shown to leech significantly less amount of bisphenol A than polysulfone or used polycarbonate bottles (Howdeshell et al. 2003), which could introduce a confounding factor. On the other hand, chlordane was dosed using sweet jelly (1% agar-agar, 0.5% sugar), due to its low solubility in water; chlordane could not be detected by GC-MS in blood samples from animals exposed to chlordane via drinking water for up to 4 weeks in pilot experiments (data not shown). The doses used for chlordane (0.05-0.5 mg/Kg/day) have been previously shown to generate plasma levels of the pesticide and its metabolite oxychlordane (1-50 ppb) similar to the ones found in the environment, including human samples (Cassidy et al. 1994). Thus, each pesticide had its own vehicle group as the routes of exposure were different among them. Pregnant rat damns were exposed to the pesticides or vehicle from gestational day 0 (G0) until weaning (PND21). Rat pups were then dosed individually (for chlordane) or in cage groups (for CPO) after weaning until the end of the experiments. A non-treated control group (naïve) was bred in parallel to be tested in some behavioral tasks and used as unfamiliar subjects for the sociability tests.

**Nissl staining**

Rats were perfused transcardially with phosphate buffer (0.1M) followed by 4% paraformaldehyde (PFA). Brains were post-fixed in PFA overnight and transferred to 30% sucrose. Coronal sections (50 μm) were cut using a vibratome (Leica) and stored in cryoprotective solution (0.2% azide in PBS). Brain tissue sections were placed in 0.5% cresyl violet in distilled water for 10-15 minutes at room temperature. Briefly, sections were sequentially rinsed in 75%, 90%, and 100% ethanol for 5 minutes before being dehydrated in xylene for 5 minutes. Sections were mounted with Permount (ThermoFisher).

For quantification of cortical thickness, a total of 4 sections (sampled every 250 μm) were used, including the somatosensory cortex. For quantification of hippocampal area, a total of 7 hippocampal sections sampled every 250 μm (from bregma –3.5 mm to bregma –5.50 mm) were used. Brain sections were scanned using ZEISS Axioscan 7 and after digitization, hippocampal area was manually outlined by using NDP2.view plus software. Total hippocampal area was calculated as the sum of individual areas from the 7 selected hippocampal sections.

**Behavior**

Tests were conducted on separate days in the following order to reduce the influence of sequential testing:

Somatic growth and righting reflex

Motor and somatic growth was evaluated from PND5 to 9 by monitoring righting reflex and body weight, respectively. Righting reflex was assessed by placing the pup on its back over a flat surface, and the time spent to return to the natural position (all four paws on the floor) was measured using a stopwatch.

Maternal homing test

Maternal homing behavior was analyzed in PND15 rats as previously described (Tonkiss et al. 1996), with minor changes. Briefly, the set-up consisted on transparent polycarbonate cage (35 × 20 × 15 cm) containing 50 ml of nest bedding (home) on one corner and an identical amount of fresh bedding on the diagonally opposite corner. The rat pup was placed in the center of the cage facing one of the empty corners, inside an opaque cylinder that was then removed to allow the animal to freely explore the arena. Each animal was videotaped over a 3-min observation period, and the time spent in each corner was manually quantified by an experimenter blind to the treatment using the AnyMaze software (Stoelting, Europe). After testing, the cage was thoroughly cleaned with 70% ethanol and tissue paper.

Hind limb clasping

Animals at weaning (PND21) were grabbed by the tail and suspended 10-15 cm above a flat surface for 10 s. Every rat was videotaped for post-testing analysis, which was performed by an experimented researcher blinded to animal treatment. Animals were given a score between 0 and 3 following a previously described scoring system (Dodge et al. 2020).

Sociability and preference for social novelty (SPSN)

Testing rats were PND27-30 and the testing arena was a Plexiglas rectangular box (65 × 55 × 35 cm). During the habituation phase, two wired cages were placed onto adjacent corners and rats were allowed to freely explore the arena for 5 min. The wired cages allow visual, auditory, olfactory interaction. In the second phase, an unfamiliar rat (gender and age-matched) was placed into one of the cages and an inanimate object (rubber duck) was placed into the other cage. The unfamiliar rats had been habituated to the wired cage for intervals of 10 minutes during 3 days prior to the test. Before testing, rats were isolated from 30 min to enhance social approach. During the test, the subject rat was placed in the center of the arena and allowed to freely explore the cages for 10 minutes. The test rat was then removed and put in a separate home cage for 5 min, while the arena was thoroughly swabbed with 70% ethanol to eliminate any olfactory cues. In the third phase, the object was replaced by a second unfamiliar rat and the testing rat was put back into the arena for another 10 min session. Behavior was recorded with a camera located above the testing box and the time the rat spent exploring the wired cage in close proximity (within 2 cm) was manually quantified by an experimenter blind to the treatment using the AnyMaze software. Results were expressed as percentage of time exploring the unfamiliar rat compared to the total time spent exploring both cages.

Juvenile social play dyad

The social play dyad was performed in PND35-36 rats as previously described (Ku et al. 2016), with modifications. In brief, unfamiliar partners were age/sex/weight matched rats housed in the same room but from different litters (naïve group), and had not previously interacted with the subject rat (they were different from the SPSN test). Before testing, rats were isolated from 30 min to enhance social approach. Rats were then placed in a rectangular box with four white opaque walls and deep bedding, and allowed to habituate to the new environment for 5 min before the unfamiliar rat was introduced. Rats were then allowed to freely interact for 10 minutes, while recorded with a camera located on top of the cage. The amount of time spent social exploring, playing and self-grooming was manually scored by an experienced investigator blinded to the treatment, based on the scoring system described by Ku and colleges (Ku et al. 2016).

Open field test

This test was used to evaluate general locomotor activity and anxiety. Five to 6 week old rats were individually placed in the center of the arena, which consisted on a Plexiglas box (65 × 55 × 35 cm) with three white opaque walls and one transparent wall, and spatial cues in two of the walls as it was later used for spatial memory tests. The animals were allowed to freely explore the arena for 10 minutes while being recorded. Their movement was tracked automatically using AnyMaze software; the arena was virtually divided into two zones, center and periphery, which were delimited at 10 cm away from each wall. Percentage of time spent in center was measured as an indication of anxiety-like behavior. Locomotor activity was analyzed using the total distance travelled across 1 min intervals.

Novel object recognition and location test (NORT/NOLT)

The tests were performed on 5-6 weeks old rats 24 h after the open field test, which was used as habituation phase for the tests. In the first phase of NORT, the rats were placed in the open field arena containing two identical objects in color, texture and shape on two adjacent corners of the arena. The rats were allowed to explore the objects for 10 minutes while being recorded. In the second phase, the re-exposure phase (24 h later), the rats were placed again in the arena containing the same objects in the same position, and were recorded for 10 min. In the test phase of NORT (24 h later), one of the objects was replaced for another one of similar dimensions and texture but different in color and shape, and again the rats were allowed to explored them for 10 min. The objects used were an orange triangular prism and a blue pyramid, and were randomly assigned as familiar or novel between the subjects to ensure they showed no preference for any of them. In the case of NOLT, the objects were rubber ducks identical in shape, dimensions and texture but slightly different in color. During the familiarization phase, the two objects were placed in the same location as the objects of NORT. In the test phase of NOLT, the location of one of the objects was changed to an opposite corner. In both NORT and NOLT, the arena and the objects were cleaned thoroughly with 70% ethanol in between phases to eliminate any olfactory cues. Behavior was recorded from a camera located above the arena and analysis was performed manually by an experimenter blinded to the treatment using the AnyMaze software. Exploration of the objects was defined as close contact sniffing (with the head pointed towards the object and within 2 cm of the object). Results were expressed as percentage of time exploring the novel object/location compared to the total time spent exploring both objects. The amount of time the animal spent exploring (head time) the empty corners was also recorder using the automatic tracking system of the AnyMaze software.

Marble burying test

The set-up consisted of a regular home cage (35 × 20 × 15 cm) with filter tops filled with 5 cm deep of fresh and compact bedding and containing 14 marbles (~ 1.5 cm diameter each) equally distributed along the walls (at 2 cm distance). Seven-week old rats were placed in the cage under bright illumination to enhance anxiety and left undisturbed for 30 min. At the end of the test, the number of marbles buried more than 2/3 of their volume were counted.

**Proteomics**

Sample preparation and Liquid chromatography Electrospray Ionization and Tandem Mass Spectrometric (LC-ESI-MS/MS) analysis

Hippocampus from 7-9 week-old adult male rats was dissected and rapidly snap-frozed at -80ºC until use. A small section of tissue from each sample was dissolved in lysis buffer containing 5% SDS, 100 mM triethylamonium bicarbonate (TEAB) and a protease/phosphatase inhibitor cocktail (ThermoFisher). Samples were reduced and alkylated by adding 5 mM tris(2-carboxyethyl)phosphine and 10 mM chloroacetamide for 30 minutes at 60ºC and homogenized by micro tip probe ultrasonication for 1 min on UP50H ultrasonic lab homogenizer (Hielscher). The homogenate was centrifuged at 16,000 × g for 15 min at 4 °C, and the supernatant containing the solubilized proteins was used for further analysis. Proteins were digested on S-Trap filters (Protifi), as described in (Ciordia et al. 2020). The resulting peptides were subsequently labelled using TMT-sixplex Isobaric Mass Tagging Kit (ThermoFisher) according to the manufacturer's instructions. A 500 ng aliquot of each digested sample was subjected to 1D-nano LC ESI-MS/MS analysis using an Ultimate 3000 nano HPLC system (ThermoFisher) coupled online to a Orbitrap Exploris 240 equipped with FAIMS Pro ion source (ThermoFisher). Peptides were eluted onto a 50 cm × 75 μm Easy‐spray PepMap C18 analytical column at 45°C and were separated at a flow rate of 300 nL/min.

Data acquisition was performed using a data-dependent top-20 method, in full scan positive mode, scanning 350 to 1200 m/z. Survey scans were acquired at a resolution of 60,000 at m/z 200, with Normalized Automatic Gain Control (AGC) target (%) of 300 and a maximum injection time of 40 ms. The top 20 most intense ions from each MS1 scan were selected and fragmented via Higher-energy collisional dissociation (HCD). Resolution for HCD spectra was set to 45,000 at m/z 200, with AGC target of 200 and maximum ion injection time of 120 ms.

Data analysis and sequence search

Raw instrument files were processed using Proteome Discoverer version 2.4 (ThermoFisher). MS2 spectra were searched using four search engines (Mascot (v2.7.0), MsAmanda (v2.4.0), MsFragger (v3.1.1) and Sequest HT) and a target/decoy database built from sequences in the *Rattus norvegicus* proteome at Uniprot Knowledgebase. The false discovery rate (FDR) for proteins, peptides, and peptide spectral matches peptides were kept at 1%. The quantification values for proteins were calculated using the abundance of total peptide for the identification of differentially expressed proteins. To calculate the p-values and adjusted p-values for quantification results, the " background-based t-test " statistical method was used. Those proteins differentially expressed with an adjusted p-value < 0.05 were considered as significant. Excel Table S1 contains information on all the proteins detected at high FRD confidence (q value < 0.01). Excel Tables S2 and S3 contain the quantification for the significant proteins in CPO and chlordane samples, respectively, as compared to vehicle. Functional analyses were performed with Ingenuity Pathway Analysis (Qiagen, Hilden, Germany).

**Bibliography**

Betancourt AM, Carr RL. The effect of chlorpyrifos and chlorpyrifos-oxon on brain, cholinesterase, muscarinic receptor binding, and neurotrophin levels in rats following early postnatal exposure. Toxicol. Sci. 2004;77(1):63–71.

Bisen-Hersh EB, Farina M, Barbosa F, Rocha JBT, Aschner M. Behavioral effects of developmental methylmercury drinking water exposure in rodents. J. Trace Elem. Med. Biol. [Internet]. NIH Public Access; 2014 Apr [cited 2016 Sep 4];28(2):117–24. Available from: http://www.ncbi.nlm.nih.gov/pubmed/24210169

Brachet A, Norwood S, Brouwers JF, Palomer E, Helms JB, Dotti CG, et al. LTP-triggered cholesterol redistribution activates Cdc42 and drives AMPA receptor synaptic delivery. J. Cell Biol. 2015;208(6):791–806.

Briz V, Baudry M. Estrogen regulates protein synthesis and actin polymerization in hippocampal neurons through different molecular mechanisms. Front. Endocrinol. (Lausanne). 2014;5(FEB):1–14.

Briz V, Restivo L, Pasciuto E, Juczewski K, Mercaldo V, Lo AC, et al. The non-coding RNA BC1 regulates experience-dependent structural plasticity and learning. Nat. Commun. [Internet]. Springer US; 2017;8(1):1–15. Available from: http://dx.doi.org/10.1038/s41467-017-00311-2

Cassidy RA, Vorhees C V., Minnema DJ, Hastings L. The effects of chlordane exposure during pre- and postnatal periods at environmentally relevant levels on sex steroid-mediated behaviors and functions in the rat. Toxicol. Appl. Pharmacol. Academic Press; 1994 Jun 1;126(2):326–37.

Ciordia S, Alvarez-Sola G, Rullán M, Urman JM, Ávila MA, Corrales FJ. Digging deeper into bile proteome. 2020 [cited 2021 Jun 15]; Available from: https://doi.org/10.1016/j.jprot.2020.103984

Dodge A, Peters MM, Greene HE, Dietrick C, Botelho R, Chung D, et al. Generation of a Novel Rat Model of Angelman Syndrome with a Complete Ube3a Gene Deletion. Autism Res. 2020;13(3):397–409.

Howdeshell KL, Peterman PH, Judy BM, Taylor JA, Orazio CE, Ruhlen RL, et al. Bisphenol A is released from used polycarbonate animal cages into water at room temperature. Environ. Health Perspect. 2003;111(9):1180–7.

Ku KM, Weir RK, Silverman JL, Berman RF, Bauman MD. Behavioral phenotyping of juvenile long-evans and sprague-dawley rats: Implications for preclinical models of autism spectrum disorders. PLoS One. 2016;11(6):1–25.

Laviola G, Adriani W, Gaudino C, Marino R, Keller F. Paradoxical effects of prenatal acetylcholinesterase blockade on neuro-behavioral development and drug-induced stereotypies in reeler mutant mice. Psychopharmacology (Berl). 2006;187(3):331–44.

Lin Z, Dodd CA, Xiao S, Krishna S, Ye X, Filipov NM. Gestational and lactational exposure to atrazine via the drinking water causes specific behavioral deficits and selectively alters monoaminergic systems in C57BL/6 mouse dams, juvenile and adult offspring. Toxicol. Sci. [Internet]. 2014 Sep [cited 2020 Apr 28];141(1):90–102. Available from: http://www.ncbi.nlm.nih.gov/pubmed/24913803

Mullen BR, Khialeeva E, Hoffman DB, Ghiani CA, Carpenter EM. Decreased Reelin Expression and Organophosphate Pesticide Exposure Alters Mouse Behaviour and Brain Morphology. ASN Neuro [Internet]. Portland Press Ltd; 2013 Jan 8 [cited 2020 Apr 22];5(1):AN20120060. Available from: http://journals.sagepub.com/doi/10.1042/AN20120060

Tonkiss J, Harrison RH, Galler JR. Differential effects of prenatal protein malnutrition and prenatal cocaine on a test of homing behavior in rat pups. Physiol. Behav. 1996;60(3):1013–8.

**Table S1. List of primary antibodies for western blotting**

| **Antibody name** | **Catalog number** | **Company name** |
| --- | --- | --- |
| P-Erk1/2 (Thr202/Tyr204) | #9106 | Cell Signaling |
| Erk1/2 | #4695 | Cell Signaling |
| P-Akt (Thr308) | #2965 | Cell Signaling |
| P-Akt (Ser473) | #4060 | Cell Signaling |
| Akt | #2920 | Cell Signaling |
| P-mTOR (Ser2448) | #5536 | Cell Signaling |
| mTOR | #4517 | Cell Signaling |
| P-S6 (Ser240/244) | #5364 | Cell Signaling |
| S6 | #2217 | Cell Signaling |
| P-p38 (T180/Y182) | #9211 | Cell Signaling |
| p38α | #9218 | Cell Signaling |
| P-S6 Kinase (Thr421/Ser424) | #9204 | Cell Signaling |
| S6 Kinase | #2708 | Cell Signaling |
| SynGAP | #3200 | Cell Signaling |
| GluA1 | AB1504 | Millipore |
| GluA2 | MAB397 | Millipore |
| GluN1 | 05-432 | Millipore |
| mGluR5 | AB5675 | Millipore |
| mGluR1 | sc-47128 | Santa Cruz |
| Homer 1 | 160-004 | Synaptic Systems |
| PSD-95 | MA1-046 | Thermo Scientific |
| α-Tubulin | T6199 | Sigma |
| GAPDH | ab8245 | Abcam |
| GFAP | G3893 | Sigma |
| Iba1 | 019-19741 | Wako |
| NKCC1 | 8351 | Cell Signaling |
| EphA3/A4/A5 | 8793 | Cell Signaling |
| VAMP2 | 13508 | Cell Signaling |
| Arp2 | sc-166103 | Santa Cruz |
| RasGRF1 | sc-377234 | Santa Cruz |
| Tau-5 | AHB0042 | Thermo Scientific |
| Calmodulin | 05-173 | Millipore |
| EAAT2 | homemade | Zafra´s Lab |

**Table S2. List of secondary antibodies for western blotting**

| **Antibody name** | **Catalog number** | **Company name** |
| --- | --- | --- |
| IRDye 680RD goat anti-mouse | P/N 925-68070 | LI-COR |
| IRDye 800CW goat anti-rabbit | P/N 925-68070 | LI-COR |
| Rabbit anti-goat HRP | A5420 | Sigma |
| Goat anti-rat HRP | 112-035-003 | Jackson |
| Donkey anti-rabbit HRP | 711-035-152 | Jackson |
| Donkey anti-mouse HRP | 715-035-151 | Jackson |
| Donkey anti-guinea pig HRP | 706-035-148 | Jackson |

**Table S3. Number of animals (N) and litters used for behavioral tests**

| **Treatment group** | **Litters** | **N total** | **N behavior** |
| --- | --- | --- | --- |
| Vehicle (drinking water) | 3 | 31 | 27 |
| CPO 0.05 mg/Kg/day | 1 | 12 | 12 |
| CPO 0.5 mg/Kg/day | 3 | 28 | 18 |
| Vehicle (sweet jelly) | 2 | 21 | 16 |
| Chlordane 0.05 mg/Kg/day | 1 | 10 | 10 |
| Chlordane 0.5 mg/Kg/day | 2 | 24 | 21 |

**Table S4. Statistical values and tests for each figure**

| **Figure** | **Test** | **P value** | **F/t value** | **Dfn** | **Dfd** |
| --- | --- | --- | --- | --- | --- |
| 1a | One-way ANOVA | 0.0004 | 3.402 | 16 | 51 |
| 1b | Two-way ANOVA (pesticides) | 0.0180 | 2.953 | 5 | 68 |
| 1b | Two-way ANOVA (AP5) | 0.3548 | 0.8679 | 1 | 68 |
| 1c | Two-way ANOVA (pesticides) | 0.0009 | 4.688 | 5 | 76 |
| 1c | Two-way ANOVA (cLTP) | <0.0001 | 118.1 | 1 | 76 |
| 1c | Two-way ANOVA (pesticides) | 0.0017 | 4.203 | 5 | 93 |
| 1c | Two-way ANOVA (DHPG) | <0.0001 | 18.47 | 1 | 93 |
| 1d | Two-way ANOVA (pesticides) | 0.2973 | 1.244 | 5 | 75 |
| 1d | Two-way ANOVA (cLTP) | <0.0001 | 23.14 | 1 | 75 |
| 1d | Two-way ANOVA (pesticides) | 0.8428 | 0.4069 | 5 | 85 |
| 1d | Two-way ANOVA (DHPG) | 0.3680 | 0.8190 | 1 | 85 |
| 1e | Two-way ANOVA (pesticides) | 0.103 | 1.952 | 5 | 48 |
| 1e | Two-way ANOVA (cLTP) | 0.8459 | 0.03816 | 1 | 48 |
| 1e | Two-way ANOVA (pesticides) | 0.0021 | 4.356 | 5 | 54 |
| 1e | Two-way ANOVA (DHPG) | 0.0007 | 12.85 | 1 | 54 |
| 1e | Two-way ANOVA (interaction) | 0.0305 | 2.690 | 5 | 54 |
| 2a | One-way ANOVA | 0.3556 | 1.112 | 3 | 40 |
| 2b | One-way ANOVA | 0.0374 | 2.500 | 5 | 78 |
| 2c | One-way ANOVA | 0.0002 | 5.509 | 5 | 70 |
| 2d | One-way ANOVA | 0.2690 | 1.348 | 4 | 40 |
| 2e | One-way ANOVA | 0.9373 | 0.06495 | 2 | 26 |
| 3a (ERK) | Two-way ANOVA (chlordane) | 0.0536 | 3.997 | 1 | 34 |
| 3a (ERK) | Two-way ANOVA (PD) | 0.0311 | 3.323 | 3 | 34 |
| 3a (ERK) | Two-way ANOVA (interaction) | 0.0011 | 6.745 | 3 | 34 |
| 3a (S6K) | Two-way ANOVA (chlordane) | 0.9247 | 0.009 | 1 | 52 |
| 3a (S6K) | Two-way ANOVA (PD) | 0.1779 | 1.702 | 3 | 52 |
| 3a (S6K) | Two-way ANOVA (interaction) | 0.0175 | 3.687 | 3 | 52 |
| 3b | Two-way ANOVA (pesticides) | 0.3771 | 1.088 | 4 | 36 |
| 3b | Two-way ANOVA (DHPG) | <0.0001 | 35.30 | 1 | 36 |
| 3c | Two-way ANOVA (pesticides) | 0.0009 | 7.747 | 2 | 71 |
| 3c | Two-way ANOVA (PD) | 0.3449 | 0.9041 | 1 | 71 |
| 4a | Two-way ANOVA (CPO) | 0.6743 | 0.1766 | 1 | 310 |
| 4a | Two-way ANOVA (chlordane) | 0.2695 | 1.224 | 1 | 300 |
| 4b | Unpaired T-test (CPO) | 0.0402 | 2.160 | - | 26 |
| 4b | Unpaired T-test (chlordane) | 0.0306 | 2.311 | - | 22 |
| 4c | Two-way ANOVA | 0.3273 | 1.120 | 2 | 419 |
| 4d | One-way ANOVA | 0.0537 | 3.119 | 2 | 46 |
| 4e | Two-way ANOVA | 0.0818 | 2.522 | 2 | 332 |
| 4f | One-way ANOVA | 0.0314 | 3.862 | 2 | 32 |
| 5a | Two-way ANOVA (CPO) | <0.0001 | 32.09 | 2 | 339 |
| 5a | Two-way ANOVA (chlordane) | <0.0001 | 37.38 | 2 | 234 |
| 5b | Two-way ANOVA (CPO) | 0.0041 | 5.721 | 2 | 135 |
| 5b | Two-way ANOVA (chlordane) | 0.6675 | 0.4061 | 2 | 87 |
| 5c | One-way ANOVA (CPO) | 0.0491 | 3.225 | 2 | 45 |
| 5c | One-way ANOVA (Chlordane) | 0.1493 | 2.032 | 2 | 29 |
| 5d | Two-way ANOVA (CPO) | <0.0001 | 19.64 | 2 | 520 |
| 5d | Two-way ANOVA (chlordane) | <0.0001 | 12.67 | 2 | 460 |
| 5e | One-way ANOVA (CPO) | 0.0407 | 3.412 | 2 | 51 |
| 5e | One-way ANOVA (chlordane) | 0.0357 | 3.611 | 2 | 42 |
| 5f | One-way ANOVA (CPO) | 0.8536 | 0.1588 | 2 | 46 |
| 5f | One-way ANOVA (chlordane) | 0.0755 | 2.828 | 2 | 29 |
| 6a | One-way ANOVA (CPO) | 0.0118 | 4.921 | 2 | 44 |
| 6a | One-way ANOVA (chlordane) | 0.2313 | 1.541 | 2 | 29 |
| 6b | One-way ANOVA (CPO) | 0.7085 | 0.3475 | 2 | 41 |
| 6b | One-way ANOVA (chlordane) | 0.1655 | 1.915 | 2 | 29 |
| 6c | Two-way ANOVA (CPO) | 0.5325 | 0.6337 | 2 | 111 |
| 6c | Two-way ANOVA (interaction) | <0.0001 | 7.633 | 4 | 111 |
| 6c | Two-way ANOVA (chlordane) | 0.7754 | 0.2551 | 2 | 87 |
| 6d (Reex) | One-way ANOVA (CPO) | 0.1844 | 1.754 | 2 | 46 |
| 6d (Reex) | One-way ANOVA (chlordane) | 0.0302 | 3.875 | 2 | 35 |
| 6d (Test) | One-way ANOVA (CPO) | 0.0417 | 3.384 | 2 | 51 |
| 6d (Test) | One-way ANOVA (chlordane) | 0.3192 | 1.176 | 2 | 39 |
| 6e | One-way ANOVA (CPO) | 0.9534 | 0.04775 | 2 | 47 |
| 6e | One-way ANOVA (chlordane) | 0.0736 | 2.801 | 2 | 37 |
| S1a | One-way ANOVA | 0.3903 | 1.085 | 16 | 56 |
| S1b | One-way ANOVA | 0.8939 | 0.5654 | 16 | 48 |
| S1c | One-way ANOVA | 0.9421 | 0.4827 | 16 | 42 |
| S1d | One-way ANOVA | 0.4176 | 1.058 | 16 | 50 |
| S2a | One-way ANOVA | 0.1866 | 1.608 | 6 | 25 |
| S2b | One-way ANOVA | 0.7303 | 0.5957 | 6 | 20 |
| S2c | One-way ANOVA | 0.0121 | 3.422 | 6 | 27 |
| S2d | One-way ANOVA | 0.0854 | 2.108 | 6 | 27 |
| S3 | Two-way ANOVA (pesticides) | 0.0002 | 11.52 | 2 | 33 |
| S3 | Two-way ANOVA (MCPG) | 0.7660 | 0.1249 | 1 | 33 |
| S4a | One-way ANOVA (ERK, Mb.) | 0.008 | 5.914 | 3 | 14 |
| S4a | One-way ANOVA (ERK, Cyt.) | 0.2111 | 1.725 | 3 | 13 |
| S4a | One-way ANOVA (p38, Mb.) | 0.1112 | 2.478 | 3 | 12 |
| S4b | One-way ANOVA (GluA1, Mb.) | 0.5049 | 0.8081 | 3 | 19 |
| S4b | One-way ANOVA (GluA1, Cyt.) | 0.3765 | 1.092 | 3 | 19 |
| S4b | One-way ANOVA (GluA2, Mb.) | 0.4765 | 0.8646 | 3 | 19 |
| S4c | One-way ANOVA (mGluR1, Mb.) | 0.2415 | 1.486 | 3 | 26 |
| S4c | One-way ANOVA (mGluR1, Cyt.) | 0.2810 | 1.422 | 3 | 13 |
| S4c | One-way ANOVA (mGluR5, Mb.) | 0.0408 | 3.160 | 3 | 27 |
| S4c | One-way ANOVA (mGluR5, Cyt.) | 0.8084 | 0.3234 | 3 | 28 |
| S4d | One-way ANOVA (GluN1, Mb.) | 0.5422 | 0.7443 | 3 | 15 |
| S4d | One-way ANOVA (Homer-1, Cyt.) | 0.3239 | 1.259 | 3 | 15 |
| S4d | One-way ANOVA (PSD-95, Mb.) | 0.8111 | 0.3195 | 3 | 17 |
| S4d | One-way ANOVA (SynGAP, Cyt.) | 0.1174 | 2.341 | 3 | 14 |
| S5a | Two-way ANOVA (pesticides) | 0.1309 | 1.785 | 5 | 56 |
| S5a | Two-way ANOVA (TEA) | <0.0001 | 89.84 | 1 | 56 |
| S5b | Two-way ANOVA (pesticides) | 0.4603 | 0.9442 | 5 | 53 |
| S5b | Two-way ANOVA (TEA) | <0.0001 | 151.8 | 1 | 53 |
| S6a | One-way ANOVA | 0.5959 | 0.6324 | 3 | 96 |
| S6b | One-way ANOVA | 0.9893 | 0,01074 | 2 | 46 |
| S6c | One-way ANOVA | 0.7330 | 0.3127 | 2 | 46 |
| S6d | One-way ANOVA | 0.0158 | 4.543 | 2 | 46 |
| S6e | One-way ANOVA | 0.4715 | 0.7642 | 2 | 46 |
| S6f | One-way ANOVA | 0.8000 | 0.2242 | 2 | 46 |
| S6g | Two-way ANOVA | <0.0001 | 22.00 | 2 | 559 |
| S6h | One-way ANOVA (amplitude) | 0.1241 | 2.186 | 2 | 45 |
| S6h | One-way ANOVA (frequency) | 0.5387 | 0.6278 | 2 | 42 |
| S7b | Mann-Whitney test | 0.8857 | 7 (U) | - | - |
| S7d | Mann-Whitney test | 0.2667 | 1 (U) | - | - |
| S7g | Mann-Whitney test | 0.1143 | 2 (U) | - | - |
| S7h | Two-way ANOVA | 0.1285 | 3.105 | 1 | 6 |
| S7i | Mann-Whitney test | 0.800 | 2 (U) | - | - |
| S7j | Two-way ANOVA | 0.5558 | 0.4369 | 1 | 3 |
| S8a | Two-way ANOVA (chlordane) | 0.3719 | 0.874 | 1 | 10 |
| S8a | Two-way ANOVA (interaction) | 0.0296 | 6.431 | 1 | 10 |
| S8a | Two-way ANOVA (CPO) | 0.0243 | 7.022 | 1 | 10 |
| S8a | Two-way ANOVA (interaction) | 0.0018 | 17.70 | 1 | 10 |
| S8b | Two-way ANOVA (chlordane) | 0.2766 | 1.324 | 1 | 10 |
| S8b | Two-way ANOVA (interaction) | 0.0031 | 14.93 | 1 | 10 |
| S8b | Two-way ANOVA (CPO) | 0.9667 | 0.0018 | 1 | 10 |
| S8b | Two-way ANOVA (interaction) | 0.2626 | 1.409 | 1 | 10 |
| S8b(Iba1) | Mann-Whitney test (chlordane) | 0.2428 | 14.5 (U) | - | - |
| S8b(Iba1) | Mann-Whitney test (CPO) | 0.0293 | 7 (U) | - | - |
| S8c | Two-way ANOVA (chlordane) | 0.5687 | 0.3472 | 1 | 10 |
| S8c | Two-way ANOVA (interaction) | 0.0449 | 5.252 | 1 | 10 |
| S8c | Two-way ANOVA (CPO) | 0.4971 | 0.4965 | 1 | 10 |
| S8c | Two-way ANOVA (interaction) | 0.0046 | 13.17 | 1 | 10 |
| S8b(Arp2) | Mann-Whitney test (chlordane) | 0.0593 | 9 (U) | - | - |
| S8b(Arp2) | Mann-Whitney test (CPO) | 0.9497 | 23 (U) | - | - |
| S8d | Two-way ANOVA (chlordane) | 0.1574 | 2.336 | 1 | 10 |
| S8d | Two-way ANOVA (interaction) | 0.0452 | 5.230 | 1 | 10 |
| S8d | Two-way ANOVA (CPO) | 0.4363 | 0.6575 | 1 | 10 |
| S8d | Two-way ANOVA (interaction) | 0.8726 | 0.027 | 1 | 10 |
| S8d(EphA) | Mann-Whitney test (chlordane) | 0.7546 | 21 (U) | - | - |
| S8d(EphA) | Mann-Whitney test (CPO) | 0.0027 | 2 (U) | - | - |
| S8e | Two-way ANOVA (chlordane) | 0.7996 | 0.0679 | 1 | 10 |
| S8e | Two-way ANOVA (interaction) | 0.0417 | 5.454 | 1 | 10 |
| S8e | Two-way ANOVA (CPO) | 0.3164 | 1.112 | 1 | 10 |
| S8e | Two-way ANOVA (interaction) | 0.1036 | 3.208 | 1 | 10 |
| S8e(CaM) | Mann-Whitney test (chlordane) | 0.9497 | 23 (U) | - | - |
| S8e(CaM) | Mann-Whitney test (CPO) | 0.4136 | 17 (U) | - | - |
| S8f (RasGRF1) | Mann-Whitney test (chlordane) | 0.1705 | 13 (U) | - | - |
| S8f (RasGRF1) | Mann-Whitney test (CPO) | 0.6620 | 20 (U) | - | - |
| S8f(Tau) | Mann-Whitney test (chlordane) | 0.2681 | 15 (U) | - | - |
| S8f(Tau) | Mann-Whitney test (CPO) | 0.6964 | 18 (U) | - | - |
| S9a | Two-way ANOVA (females) | 0.0234 | 3.950 | 2 | 75 |
| S9a | Two-way ANOVA (males) | 0.0314 | 3.723 | 2 | 48 |
| S9b | One-way ANOVA (females) | 0.2791 | 1.343 | 2 | 25 |
| S9b | One-way ANOVA (males) | 0.0659 | 3.206 | 2 | 17 |
| S11a | One-way ANOVA (CPO) | 0.0989 | 2.508 | 2 | 29 |
| S11a | One-way ANOVA (chlordane) | 0.0496 | 3.395 | 2 | 25 |
| S11b | One-way ANOVA (CPO, females) | 0.6649 | 0.4154 | 2 | 23 |
| S11b | One-way ANOVA (CPO, males) | 0.4137 | 0.9367 | 2 | 15 |
| S11b | One-way ANOVA (chlordane, females) | 0.5239 | 0.6733 | 2 | 16 |
| S11b | One-way ANOVA (chlordane, males) | 0.0373 | 4.653 | 2 | 10 |
| S12a | One-way ANOVA (CPO) | 0.0639 | 2.922 | 2 | 46 |
| S12a | One-way ANOVA (chlordane) | 0.2002 | 1.673 | 2 | 41 |
| S12b | One-way ANOVA (CPO, old) | 0.0492 | 3.347 | 2 | 29 |
| S11b | One-way ANOVA (CPO, neutral) | 0.5050 | 0.7008 | 2 | 27 |
| S11b | One-way ANOVA (chlordane, old) | 0.9300 | 0.07280 | 2 | 28 |
| S11b | One-way ANOVA (chlordane, neutral) | 0.0743 | 2.857 | 2 | 28 |
